# Supplementary material for: Effect of sequence depth and length in long-read assembly of the maize inbred NC358
Source: Nat Commun. 2020 May 8;11:2288. doi: 10.1038/s41467-020-16037-7 (PMC7211024; doi:10.1038/s41467-020-16037-7)
Supplement: Supplementary file 1 — Supplementary Information [file 41467_2020_16037_MOESM1_ESM.pdf]

Effect of sequence depth and length in long-read assembly of the maize inbred NC358

Ou *et al.*

Supplementary Note 1. Parameter for FALCON genome assembly

FALCON only assembly for 75-fold NC358:
pa_HPCdaligner_option = -k14 -e0.75 -s100 -l3000 -h240 -w8 -H14154
ovlp_HPCdaligner_option = -k24 -e.95 -s100 -l1000 -h600 -H20149
pa_DBsplit_option = -x500 -s400
ovlp_DBsplit_option = -x500 -s400
falcon_sense_option = --min_idt 0.70 --min_cov 2 --max_n_read 200
overlap_filtering_setting = --max_diff 40 --max_cov 80 --min_cov 2 --min_len 500

Canu only assembly for 75-fold NC358:
ovlMerThreshold=500; genome_size=2272400000; input_type=-pacbio-raw

WTDBG2 assembly for 75-fold NC358:
-x sq -g 2272400000 -t128

FALCON-Peregrine hybrid assembly for 75-fold NC358:
FALCON
pa_HPCdaligner_option = -k14 -e0.75 -s100 -l3000 -h240 -w8 -H14154
pa_DBsplit_option = -x500 -s400
falcon_sense_option = --min_idt 0.70 --min_cov 2 --max_n_read 200

Peregrine
find /wd/top24x/ -name "*.fa" | sort > reads.lst
docker run -it -v /wd:/wd cschin/peregrine:latest asm /wd/reads.lst 24 24 24 12 24 24 24 24 24 --with-consensus --shimmer-w 80 --shimmer-r 3 --best_n_ovlp 24 --mc_upper 640 --output /wd/top24x_asm/

FALCON-Flye hybrid assembly for 75-fold NC358:
FALCON
pa_HPCdaligner_option = -k14 -e0.75 -s100 -l3000 -h240 -w8 -H14154
pa_DBsplit_option = -x500 -s400
falcon_sense_option = --min_idt 0.70 --min_cov 2 --max_n_read 200
Flye
--pacbio-corr nc358.merged.fa.gz --genome-size 2272000000

FALCON-Canu hybrid assembly for 75-fold NC358:
FALCON
pa_HPCdaligner_option = -k14 -e0.75 -s100 -l3000 -h240 -w8 -H14154
pa_DBsplit_option = -x500 -s400

falcon_sense_option = --min_idt 0.70 --min_cov 2 --max_n_read 200
CANU
ovlMerThreshold=500; genome_size=2272400000; input_type=-pacbio-corrected

FALCON-Canu hybrid assembly for 60-fold NC358:
FALCON
pa_HPCdaligner_option = -k14 -e0.75 -s100 -l3000 -h240 -w8 -H10310
pa_DBsplit_option = -x500 -s400
falcon_sense_option = --min_idt 0.70 --min_cov 2 --max_n_read 200
CANU
ovlMerThreshold=500; genome_size=2272400000; input_type=-pacbio-corrected

FALCON-Canu hybrid assembly for 50, 40, 30, 20-fold NC358:
FALCON
pa_HPCdaligner_option = -k14 -e0.75 -s100 -l3000 -h240 -w8 -H3000
pa_DBsplit_option = -x500 -s400
falcon_sense_option = --min_idt 0.70 --min_cov 2 --max_n_read 200
CANU
ovlMerThreshold=500; genome_size=2272400000; input_type=-pacbio-corrected

FALCON-Canu hybrid assembly for 50-fold NC358 with shifted distribution (11 kb and 16 kb):
FALCON
pa_HPCdaligner_option = -k14 -e0.75 -s100 -l3000 -h240 -w8 -H3000
pa_DBsplit_option = -x500 -s400
falcon_sense_option = --min_idt 0.70 --min_cov 2 --max_n_read 200
CANU
ovlMerThreshold=500; genome_size=2272400000; input_type=-pacbio-corrected

FALCON only assembly for 68-fold B73:
pa_HPCdaligner_option = -k14 -e0.75 -s100 -l3000 -h240 -w8 -H9898
ovlp_HPCdaligner_option = -k29 -e.95 -s100 -l4800 -h600 -H12360
pa_DBsplit_option = -x500 -s400
ovlp_DBsplit_option = -x500 -s400
falcon_sense_option = --min_idt 0.70 --min_cov 2 --max_n_read 200
overlap_filtering_setting = --max_diff 40 --max_cov 80 --min_cov 2 --min_len 500

Canu only assembly for 68-fold B73:
ovlMerThreshold=500; genome_size=2500000000; input_type=-pacbio-raw

FALCON-Canu hybrid assembly for 68-fold B73:

FALCON
pa_HPCdaligner_option = -k14 -e0.75 -s100 -l3000 -h240 -w8 -H9898
pa_DBsplit_option = -x500 -s400
falcon_sense_option = --min_idt 0.70 --min_cov 2 --max_n_read 200
CANU
ovlMerThreshold=500; genome_size=2272400000; input_type=-pacbio-corrected

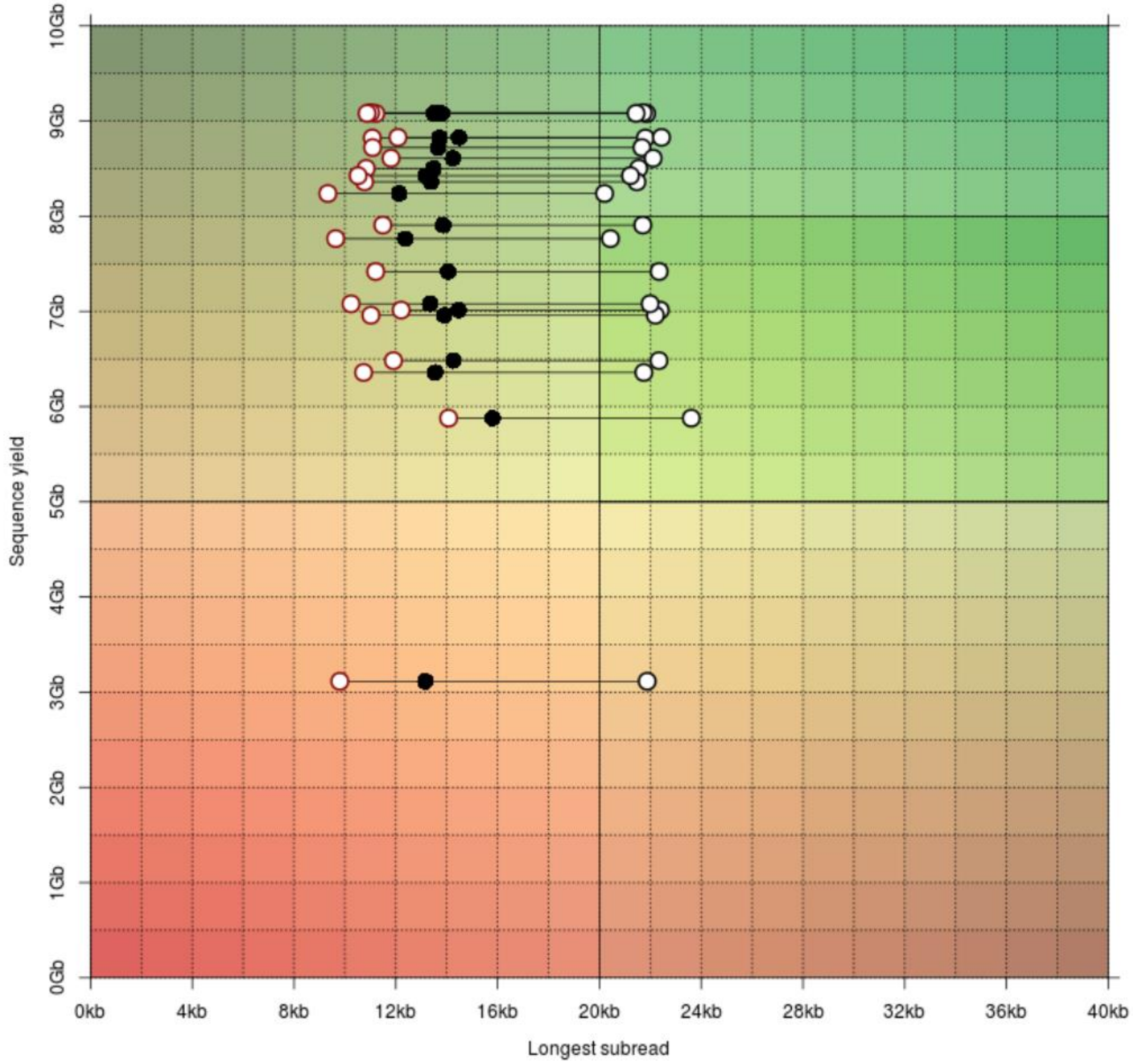

Supplementary Figure 1. Statistics for NC358 subreads derived from 21 SMRT cells. X-axis, subread length. For a continuous long read (CLR) with multiple passes, only the longest subread was used. Y-axis, sequence yield. Each line represents a SMRT cell with black dots, white dots with red outlines, and white dots with black outlines represent subread mean, median, and N50 lengths, respectively.

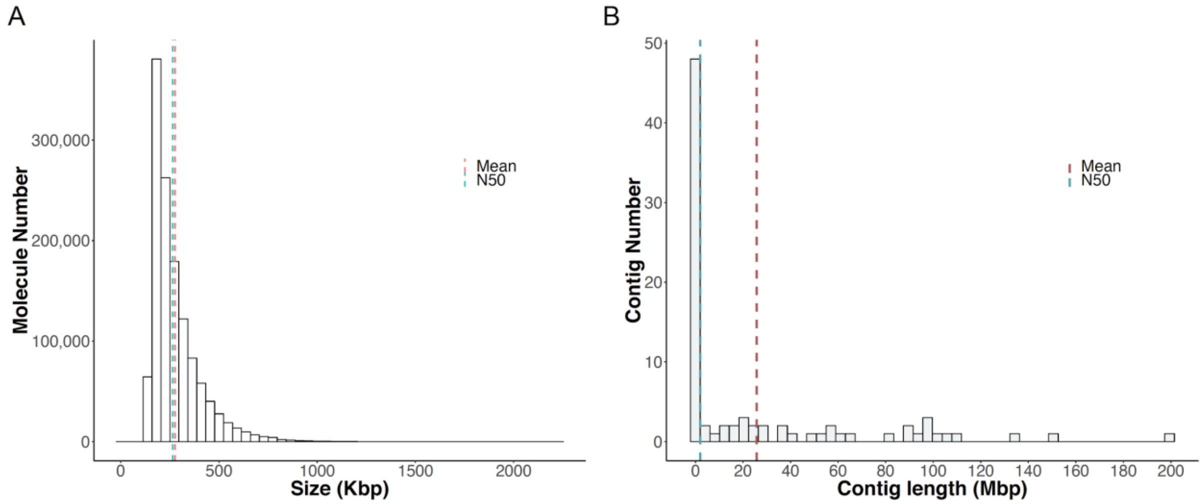

Supplementary Figure 2. Summary of the NC358 Bionano optical map. (A) BNX molecule length distribution. (B) Bionano contig length distribution. Source data are provided as a Source Data file.

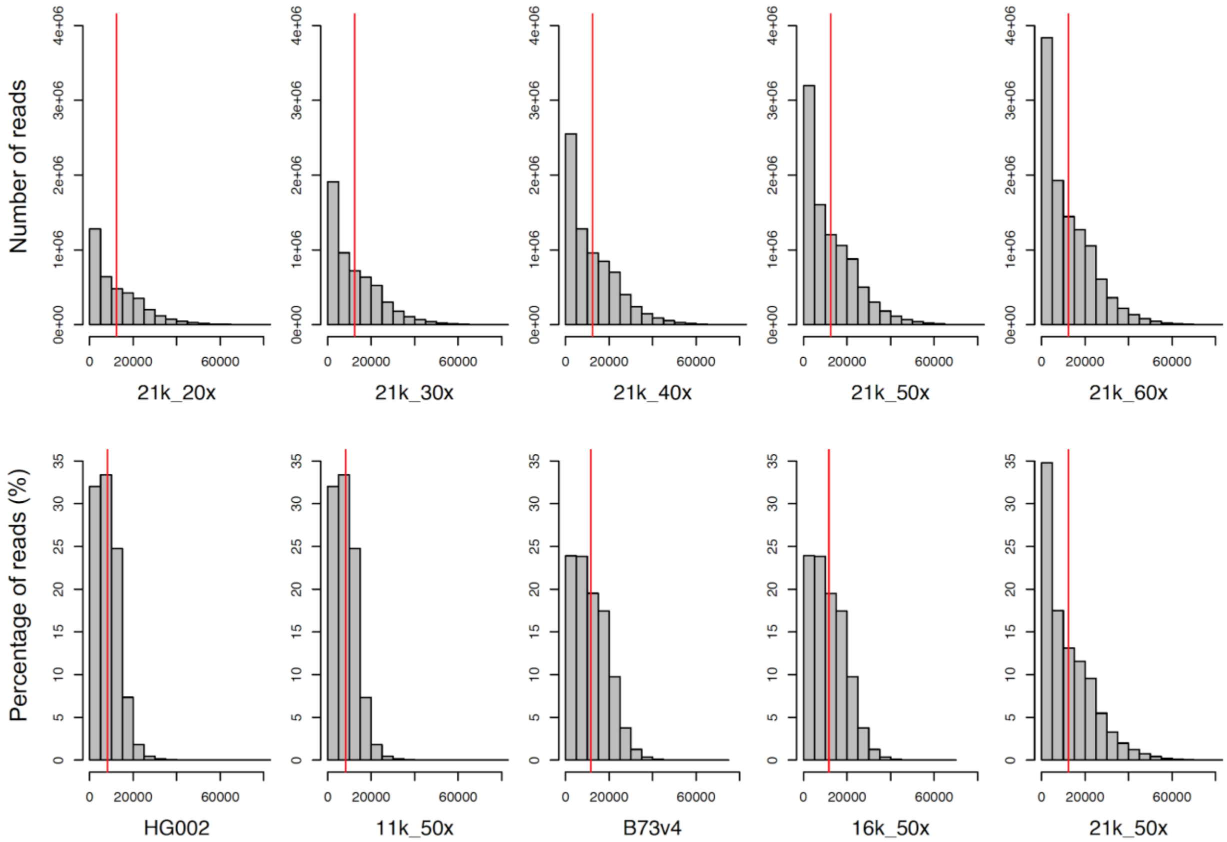

Supplementary Figure 3. Subread length distributions of different datasets. X-axes, read length in bp. Top row, read count was presented. Bottom row, read density was presented. Red vertical line, mean read length of the dataset.

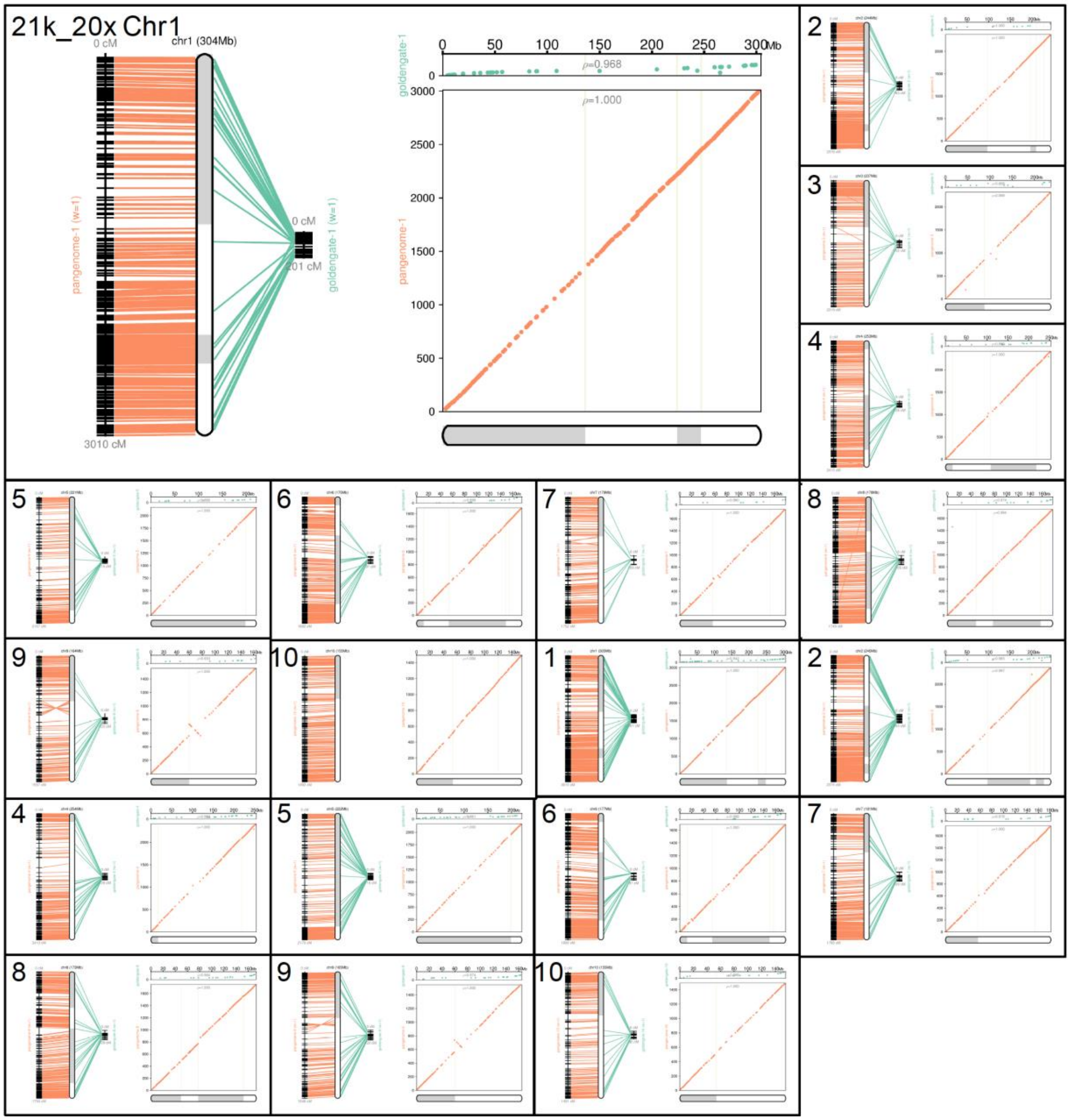

Supplementary Figure 4. Construction of pseudomolecules using both GoldenGate and Pangenome markers. The NC358 21k_20x and 21k_75x are shown on the top and bottom, respectively, with each chromosome indicated on a single subplot. The 21k_75x chromosome 3 consists of a single scaffold, thus no pseudomolecule construction is needed and therefore not shown in the figure. Orange lines and dots, pangenome markers mapped to the pseudomolecule; Green lines and dots, GoldenGate markers mapped to the pseudomolecule; Grey and white regions, different superscaffolds of the pseudomolecule. Plots for the rest of the NC358 assemblies are available here: https://github.com/HuffordLab/Maize_NC358/tree/master/AGP/AGP_plots. Source data are provided as a Source Data file.

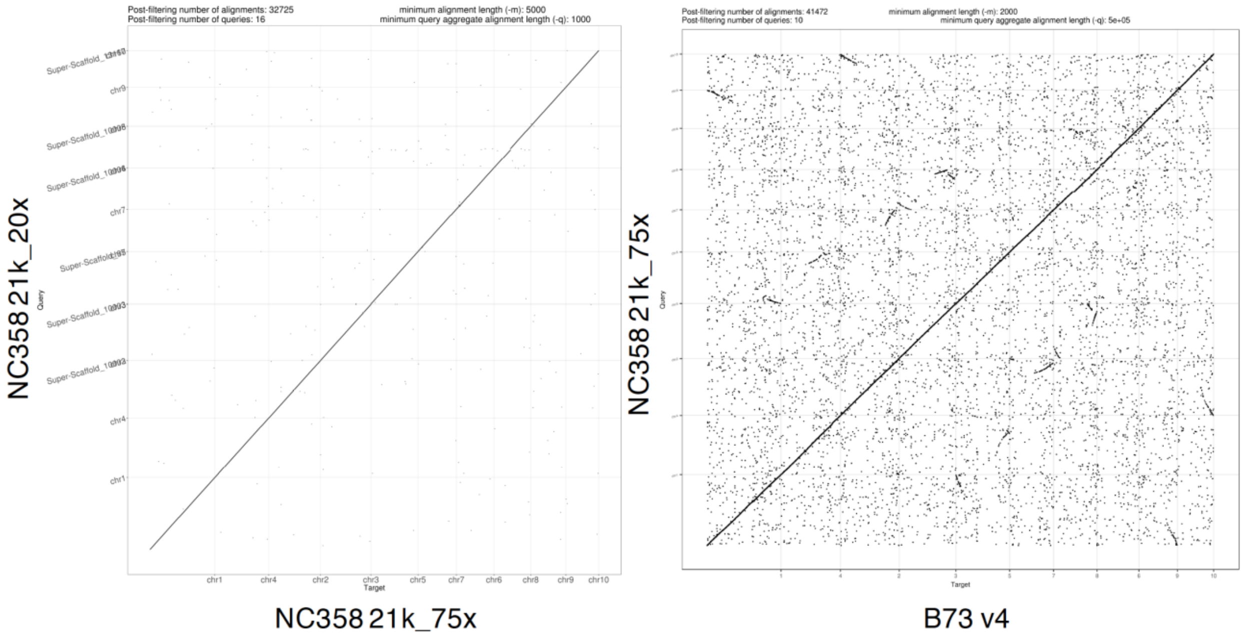

Supplementary Figure 5. Synteny between NC358 21k_20x, NC358 21k_75x, and B73 v4 genomes.

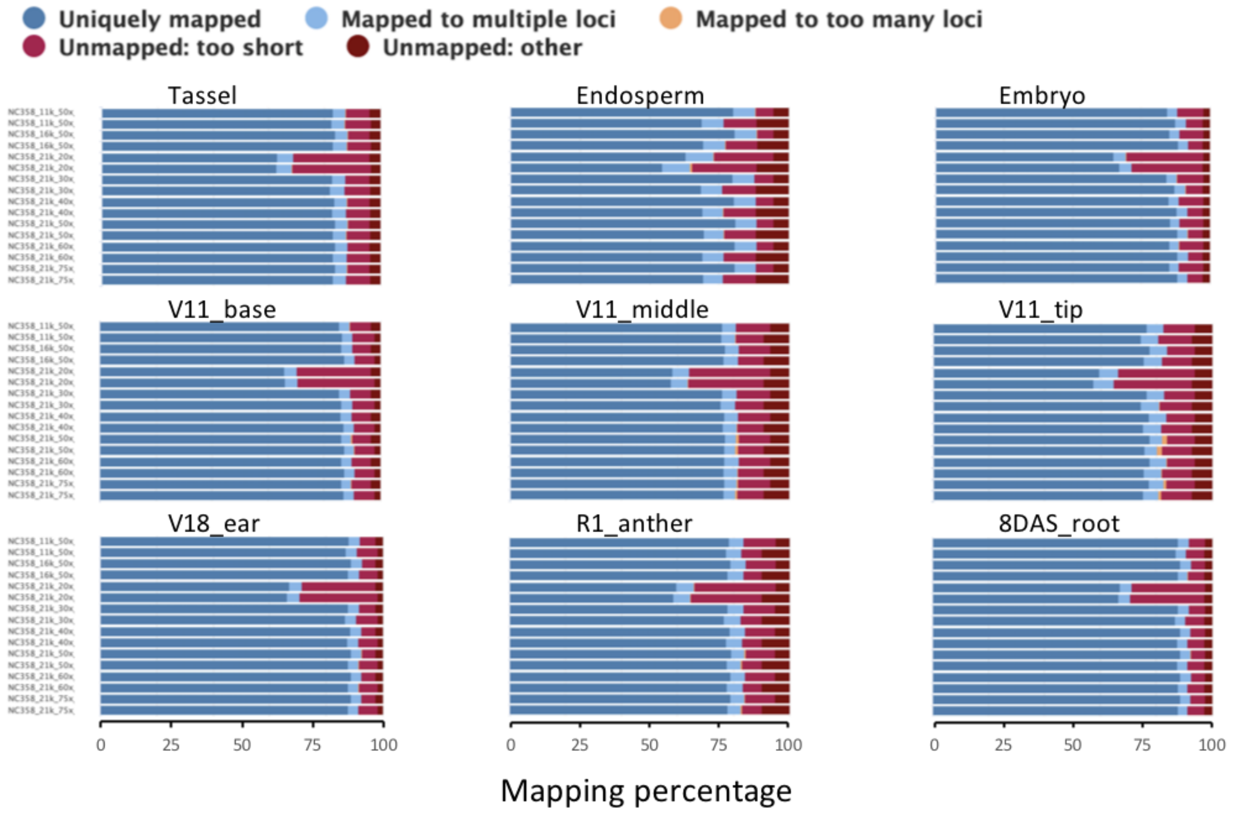

Supplementary Figure 6. RNA-seq mapping of nine tissues to various NC358 assemblies. Each replicate (total two for each tissue) was mapped independently. The 21k_20x assembly showed significantly low RNA-seq mapping rates. Source data are provided as a Source Data file.

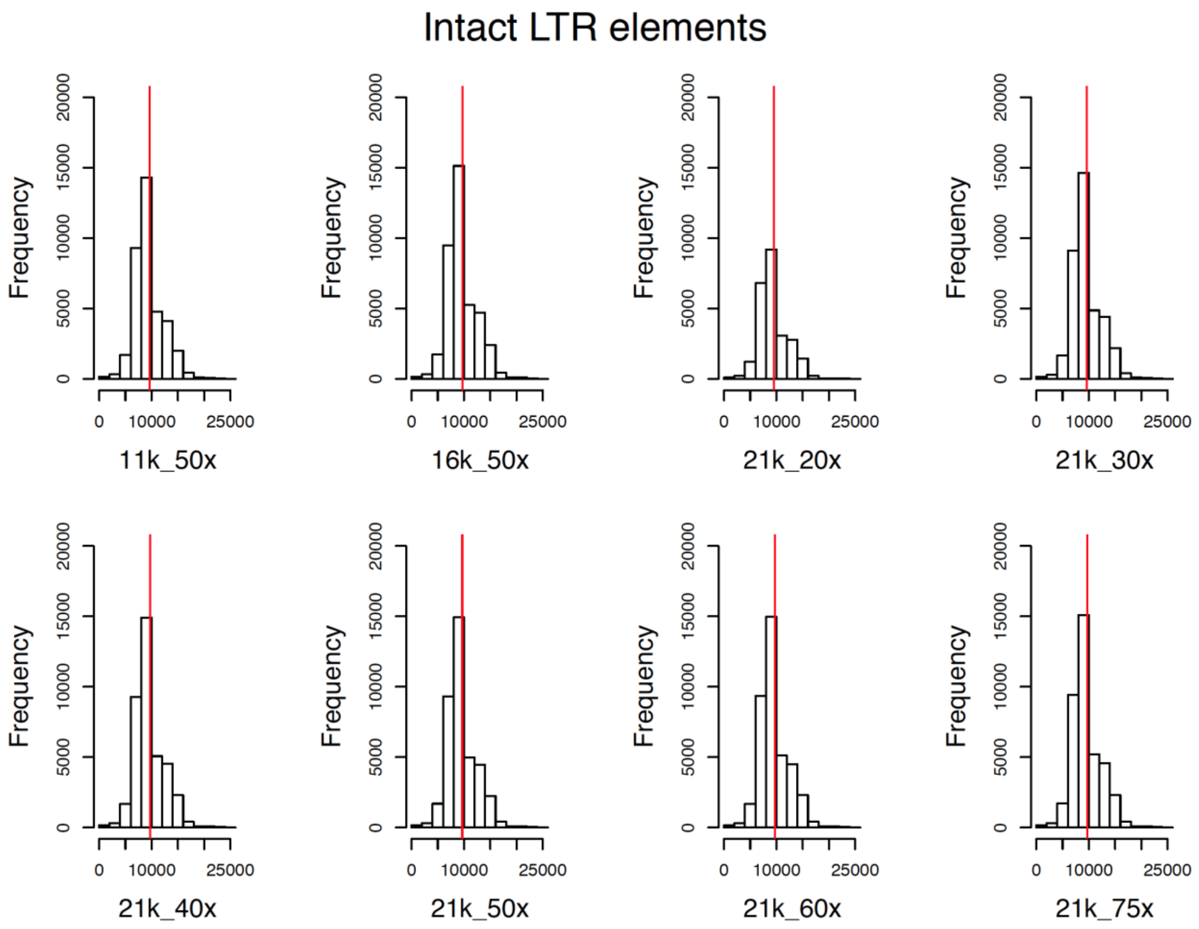

Supplementary Figure 7. Length distribution of intact long terminal repeat retrotransposons in NC358 assemblies. X-axes, length of LTR retrotransposons. Y-axes, count of LTR retrotransposons. Red lines, mean length of long terminal repeat (LTR) retrotransposons. Source data are provided as a Source Data file.

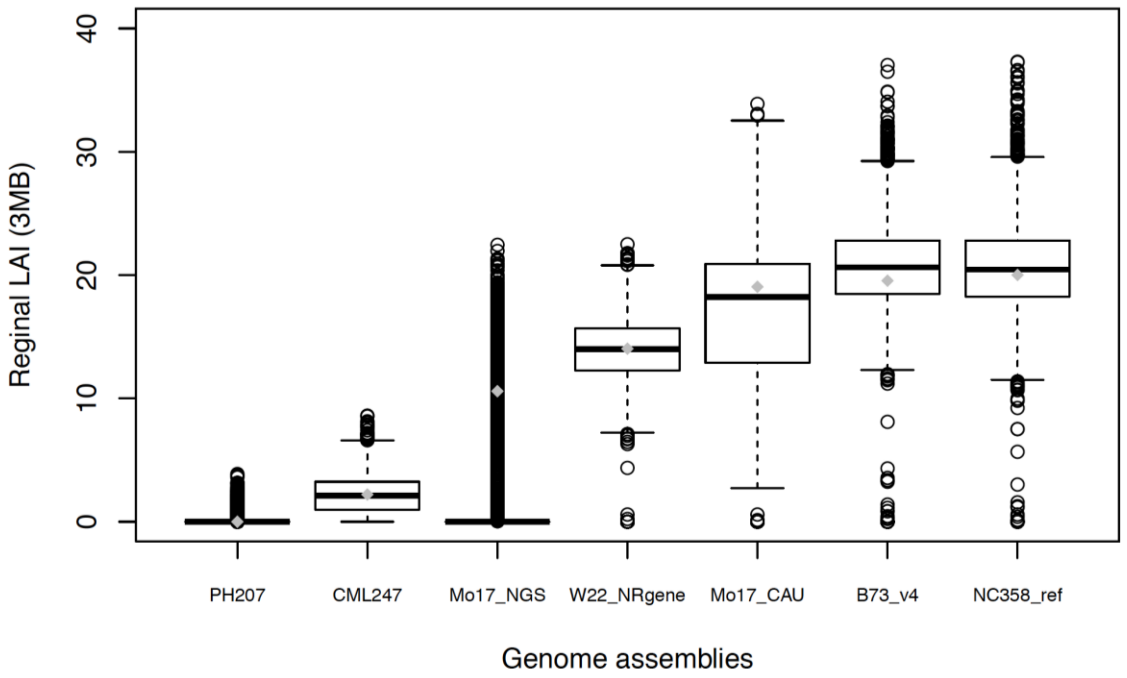

Supplementary Figure 8. LTR assembly index of NC358 and published genomes. Grey dot, whole-genome LTR assembly index (LAI). The box shows the median, upper and lower quartiles. Whiskers indicate values ≤ 1.5× interquartile range. Outliers are plotted as circles. Source data are provided as a Source Data file.

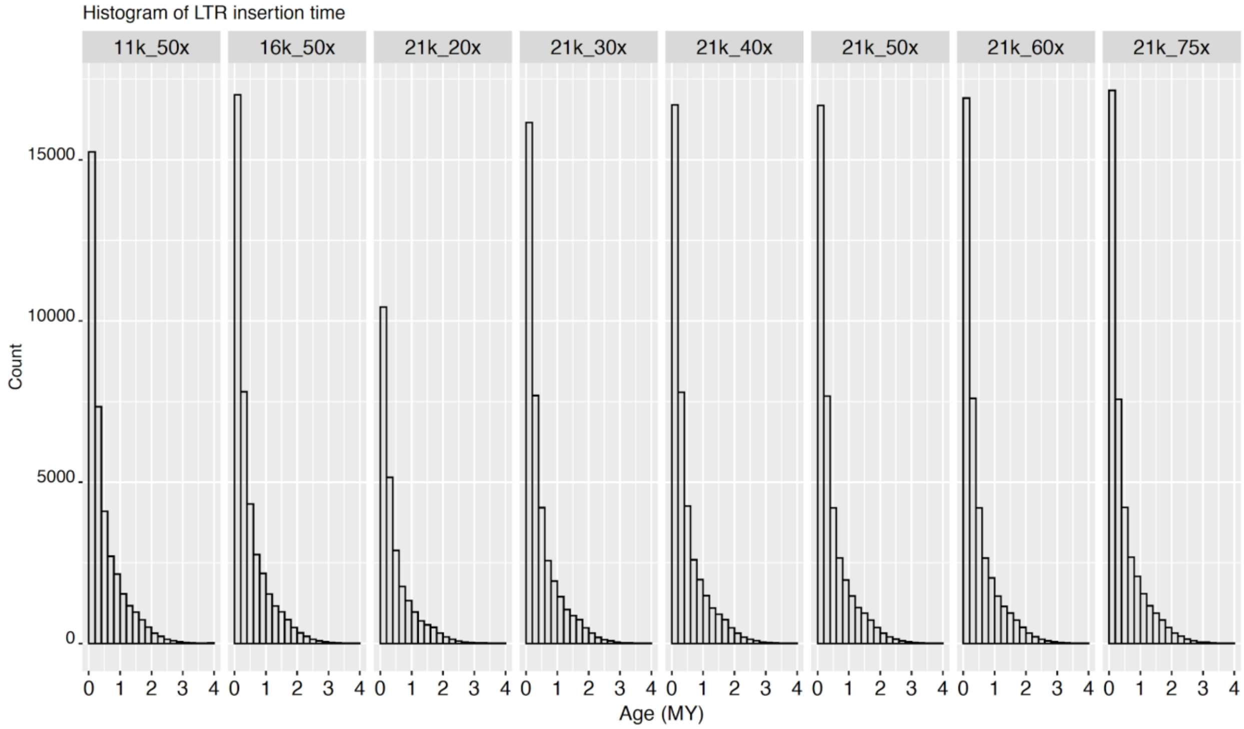

Supplementary Figure 9. Distribution of insertion time of intact long terminal repeat retrotransposons identified in NC358 assemblies. LTR: long terminal repeat. Source data are provided as a Source Data file.

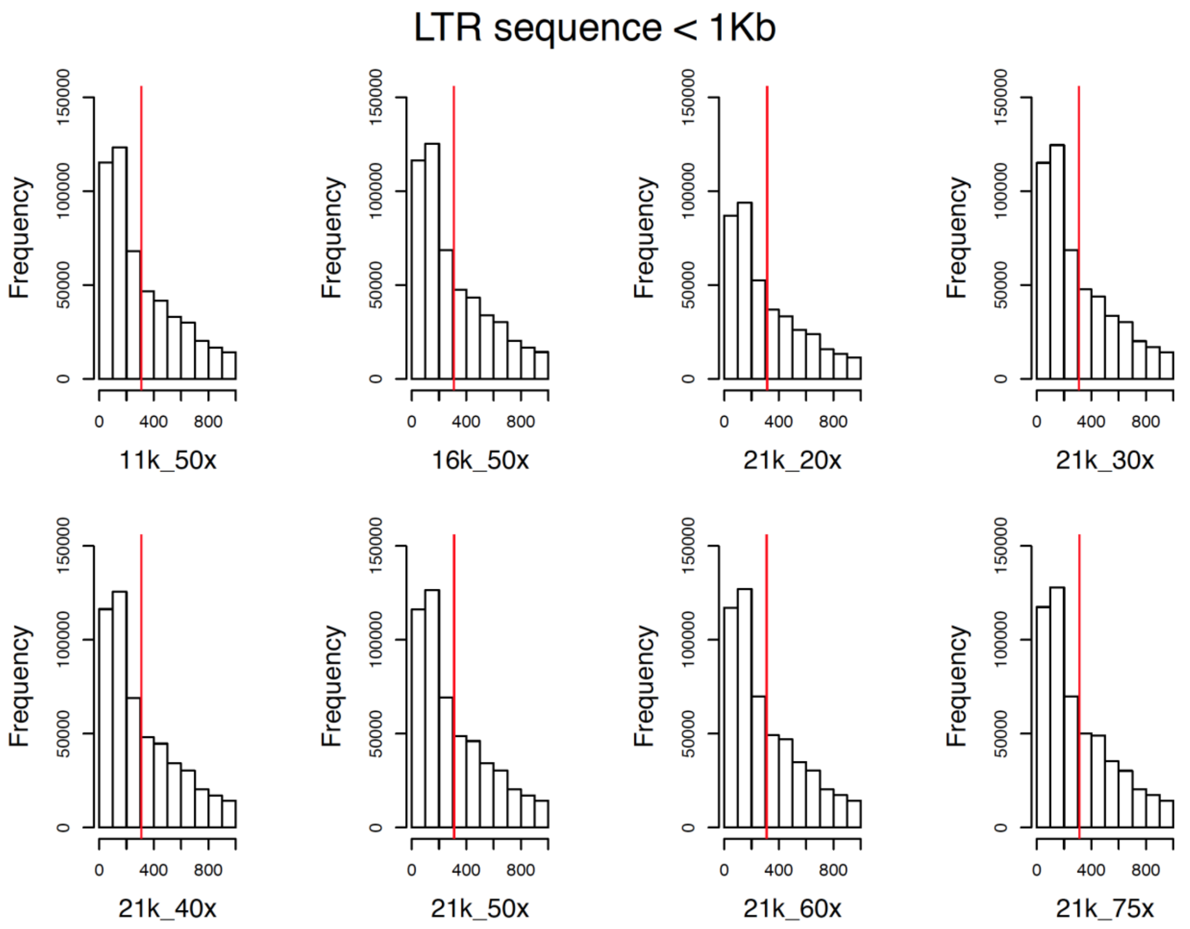

Supplementary Figure 10. Length distribution of fragmented long terminal repeat retrotransposons shorter than 1 kb in NC358 assemblies. X-axes, length of LTR fragments. Y-axes, count of LTR retrotransposons. Red lines, mean length of long terminal repeat (LTR) retrotransposons. Source data are provided as a Source Data file.

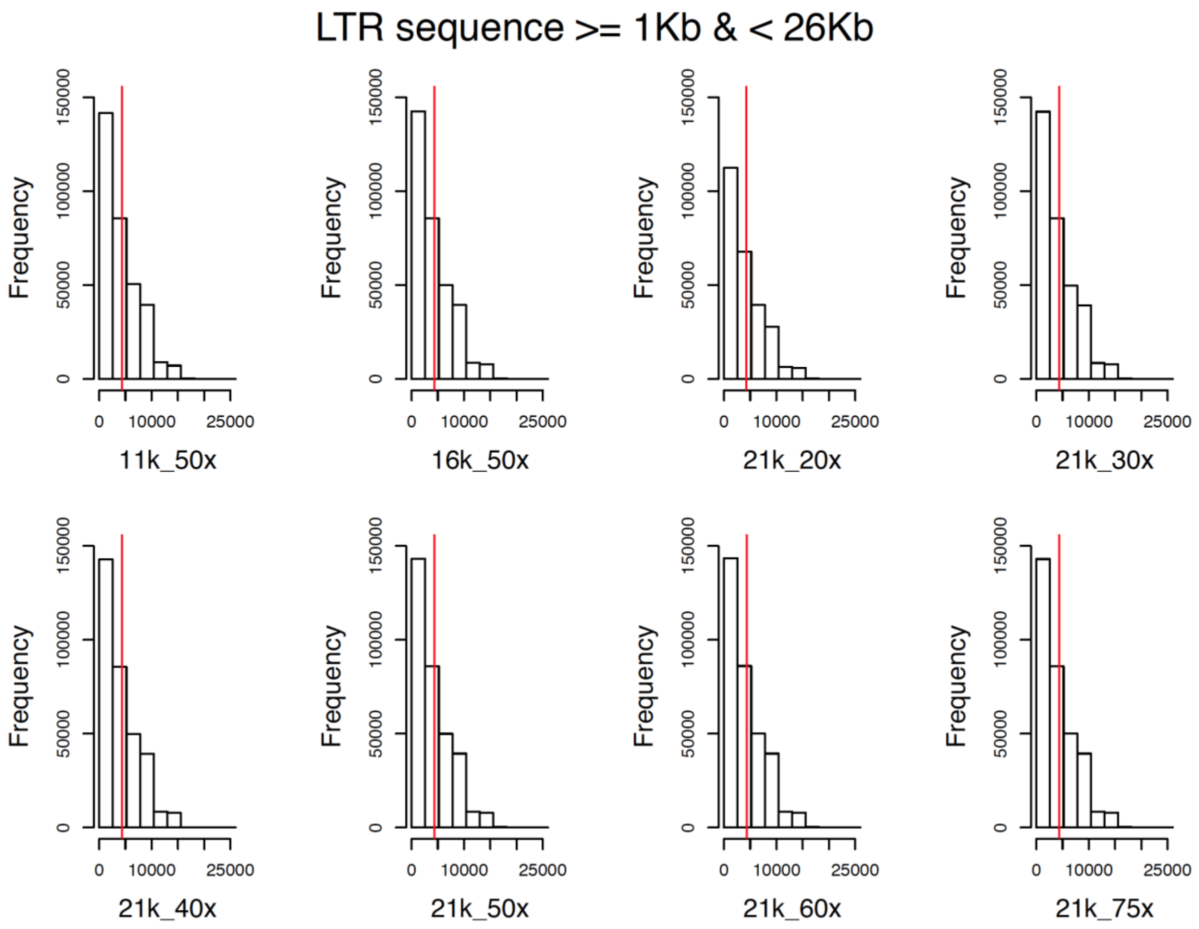

Supplementary Figure 11. Length distribution of fragmented long terminal repeat retrotransposons ranging from 1 kb to 26 kb in NC358 assemblies. X-axes, length of long terminal repeat (LTR) fragments. Y-axes, count of LTR retrotransposons. Red lines, mean length of LTR retrotransposons. Source data are provided as a Source Data file.

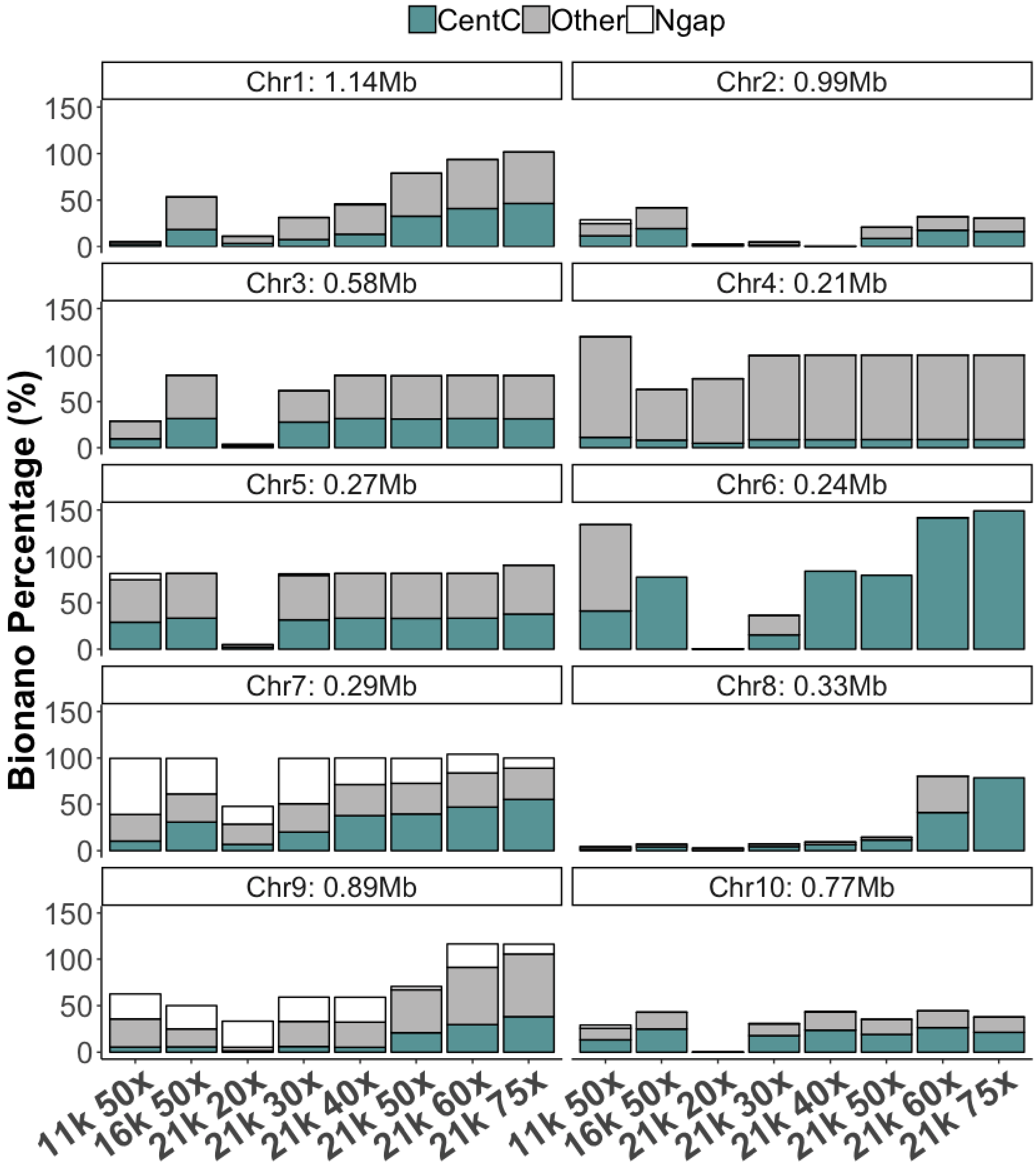

Supplementary Figure 12. Evaluation of CentC array assembly using the bionano optical map. CentC, CentC array found in the ChIP-defined centromere region. Ngap, non-13N gaps. Other, other sequence contents.

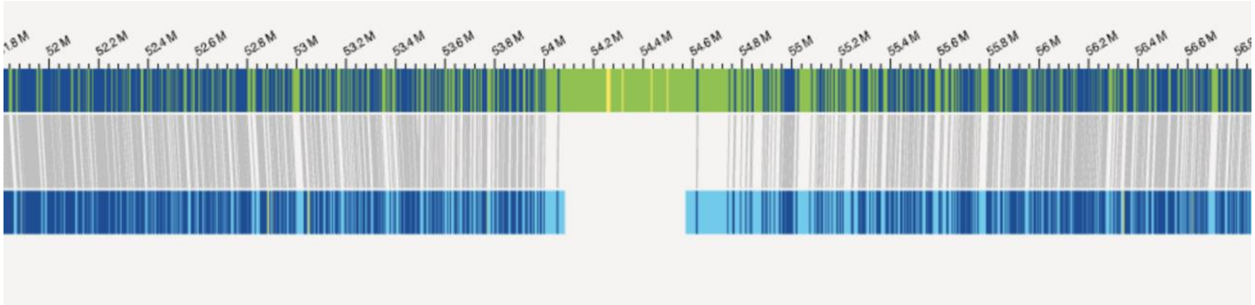

Supplementary Figure 13. An example of failure in Bionano *de novo* assembly due to lack of DLE-1 label in CentC regions, where sequence assembly could be more contiguous. The upper panel represents the reference genome, which is the pseudomolecule chr6 in the 21k_75x assembly. The lower panels are the Bionano *de novo* assembled optical maps used as queries. The blue bars indicate the matching label sites between reference and query, while yellow bars represent unmatched sites.

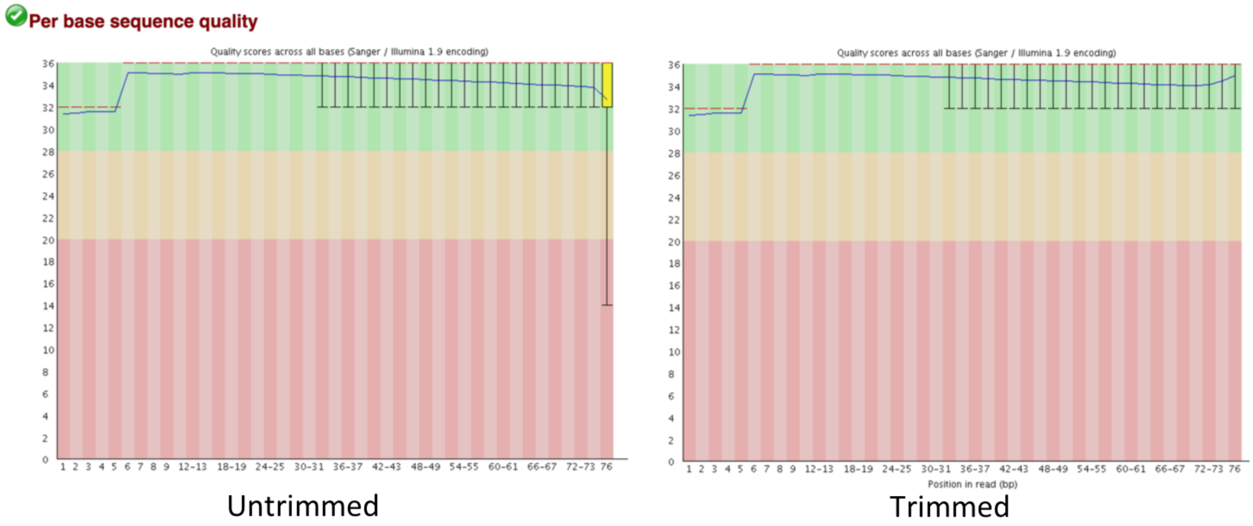

Supplementary Figure 14. An example of RNA-seq libraries for quality trimming. X-axes, base pairs on RNA-seq reads. Y-axes, Phred-scaled quality scores. The box shows the median (red line), upper and lower quartiles. The blue line shows the mean. The upper and lower whiskers indicate the 10% to 90% range.

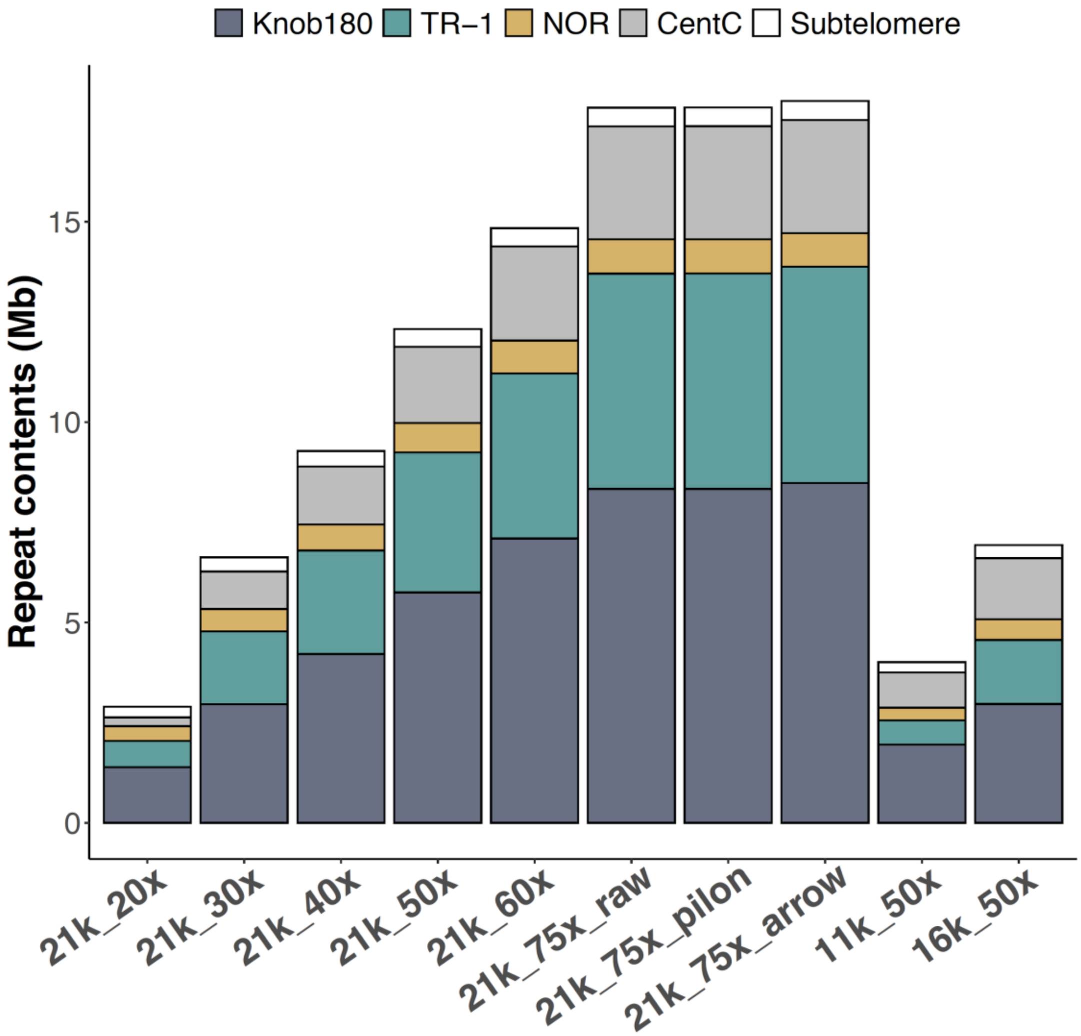

Supplementary Figure 15. Assembled repeat contents in NC358 assemblies. The 21k_75x_pilon assembly was polished using Pilon with 30x Illumina short reads. The 21k_75x_arrow assembly was polished using Arrow with the full 75x PacBio dataset. The rest of NC358 assemblies are non-polished. Source data are provided as a Source Data file.

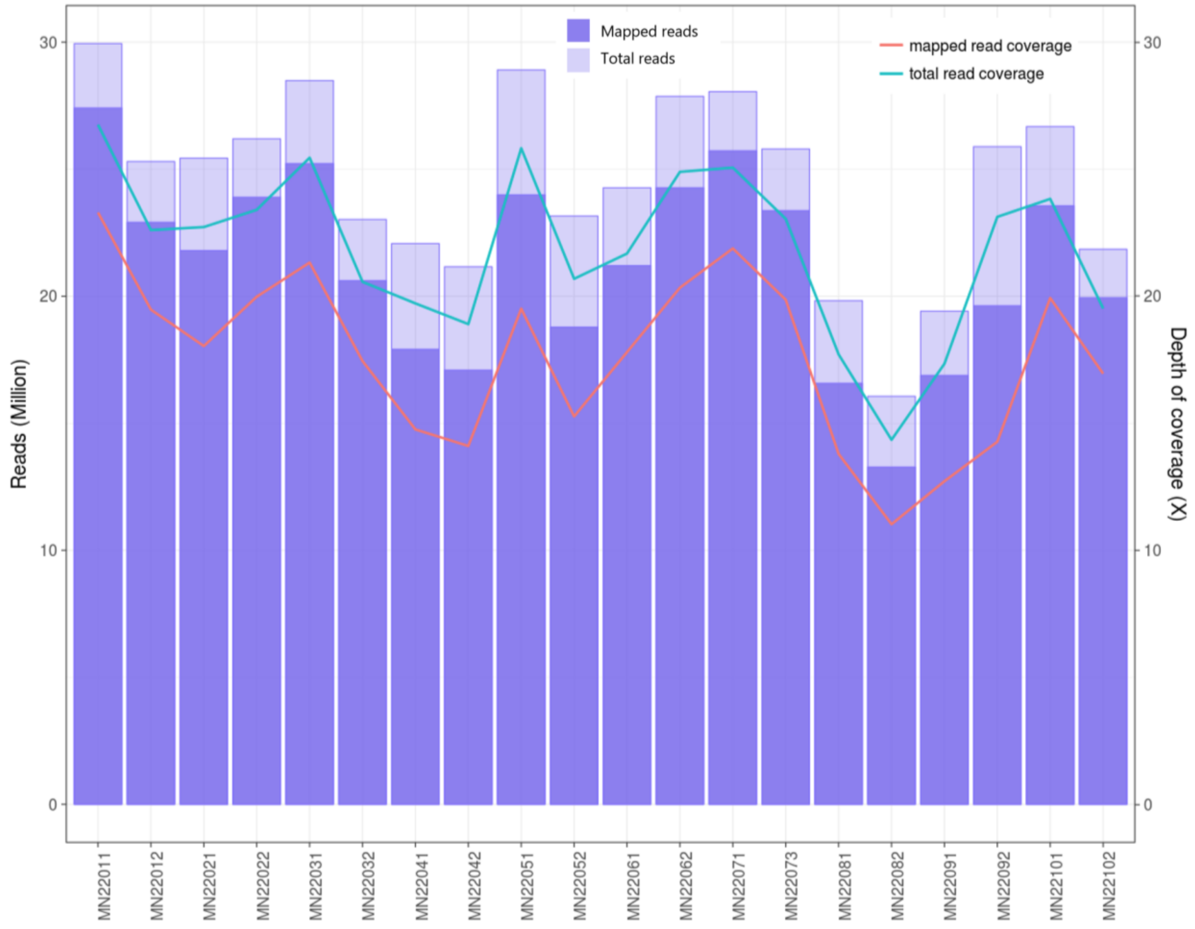

Supplementary Figure 16. Quality checks of NC358 RNA-seq libraries. RNA sequences were trimmed and mapped to the B73 v4 genome. Mapped read coverage refers to coverage from both unique and multiple mapped reads. Read coverage was calculated using a total CDS length of 266 MB. MN22031, V11 base; MN22032, V11 base; MN22021, 8DAS shoot; MN22022, 8DAS shoot; MN22062, V18 tassel; MN22061, V18 tassel; MN22051, V11 tip; MN22052, V11 tip; MN22041, V11 middle; MN22042, V11 middle; MN22081, R1 anther; MN22082, R1 anther; MN22102, 16DAP embryo; MN22101, 16DAP embryo; MN22073, V18 ear; MN22071, V18 ear; MN22012, 8DAS root; MN22011, 8DAS root; MN22092, 16DAP endosperm; and MN22091, 16DAP endosperm. Source data are provided as a Source Data file.

Supplementary Table 1. Summary statistics of SMRT sequencing data.

No. of SMRT cells		21
Sequencing Center		Arizona Genomics Institute
	Average Length	12,403
	Average N50	21,196
	Total bases (Gb)	171
	Average Gb/cell	8
Subreads	Coverage	75

Supplementary Table 2. Selection of assembly scheme.

	B73	B73	B73	NC358	NC358	NC358	NC358	NC358	NC358	NC358	NC358
Experiment	Falcon	Canu	Falcon-Canu hybrid	Falcon	Canu	Falcon-Canu hybrid	Falcon-Canu hybrid + Arrow	Falcon-Canu hybrid + Pilon	WTDBG2	Falcon-Peregrine hybrid top23x	Falcon-Flye hybrid
Raw data base pair	156,402,786,403	156,402,786,403	156,402,786,403	171,084,765,118	171,084,765,118	171,084,765,118	171,084,765,118	171,084,765,118	171,084,765,118	171,084,765,118	171,084,765,118
Raw data coverage	68	68	68	75	75	75	75	75	75	75	75
Raw data N50	16,765	16,765	16,765	21,166	21,166	21,166	21,166	21,166	21,166	21,166	21,166
Error corrected base pair	80,011,532,822	85,303,967,835	80,011,532,822	100,900,661,550	91,385,104,775	100,900,661,550	100,900,661,550	100,900,661,550	-	52,003,348,779	100,900,661,550
Error corrected coverage	35	37	35	44	40	44	44	44	-	22.6	44
Error corrected N50	14,401	15,784	14,401	20,054	22,401	20,054	20,054	20,054	-	26,836	20,054
Contig total bp	2,091,596,294	2,113,638,982	2,116,302,017	2,103,976,804	2,120,369,996	2,128,806,389	2,133,480,938	2,130,838,090	3,430,000,000	2,628,817,360	2,137,995,282
Percent assembly	92.0%	93.0%	94.0%	92.6%	93.3%	93.7%	93.9%	93.8%	151.1%	115.7%	94.1%
Contig NG50	2,506,659	1,747,454	2,981,971	3,475,790	7,800,781	22,986,710	23,030,234	23,006,897	160,723	12,986,327	2,128,602
Contig N50	2,701,304	1,907,435	3,308,794	3,659,773	8,795,983	24,541,753	24,589,835	24,562,153	101,071	10,973,664	2,260,985
Contig N90	842,700	553,169	928,165	1,140,198	2,269,078	5,209,146	5,214,652	5,211,235	22,413	59,531	532,077
Max contig size	10,181,848	7,495,811	19,745,572	13,771,434	61,269,762	78,424,123	78,731,825	78,584,751	1,102,694	54,516,146	10,205,862
N.contig	1,296	2,014	1,266	1,012	571	327	328	327	65,901	9,316	4,156
Complete BUSCOs (%)	88.3	95.7	94.9	78.4	88.0	91.7	96.3	96.3	72.4	95.2	96.4
Complete & single copy BUSCOs (%)	84	90.4	89.7	75.8	84.2	87.5	91.0	91.3	69.1	90.3	91.0
Conflict with DLE-1	370	121	64	137	34	21	21	21	1,691	135	321
LAI	17.2	21.0	21.1	17.0	21.0	20.6	21.6	20.9	2.5	19.7	21.0

Supplementary Table 3. Statistics of assembly variants detected by optical map*.

Assembly	Type	Count	Mininum (bp)	Mean (bp)	Median (bp)	Maximum (bp)
21k_20x	insertion	880	502	16129	14702	77639
21k_20x	deletion	145	500	21957	7297	233871
21k_30x	insertion	785	509	14474	10992	490311
21k_30x	deletion	156	535	45109	4666	4078301
21k_40x	insertion	325	507	18379	11985	859979
21k_40x	deletion	134	507	22275	4342	407617
21k_50x	insertion	215	501	17056	12792	130157
21k_50x	deletion	118	559	25327	5172	234431
21k_60x	insertion	217	511	15004	9643	171212
21k_60x	deletion	86	514	28463	4132	340624
21k_75x	insertion	113	509	3813	978	28741
21k_75x	deletion	60	506	19512	7094	165740
11k_50x	insertion	3958	517	8908	8527	109369
11k_50x	deletion	241	522	17711	4089	339667
16k_50x	insertion	687	500	12000	10994	74548
16k_50x	deletion	155	507	20689	4724	247502

*Source data are provided as a Source Data file.

Supplementary Table 4. Statistics on markers used for ordering and orientation of scaffolds.

Assembly	Mapped GoldenGate	Mapped Pangenome	Placed (bp)	Placed (%)	Unplaced (bp)	Unplaced (%)
11k_50x	343	3,234	2,143,236,220	99.80%	4,577,001	0.20%
16k_50x	353	3,132	2,121,376,533	99.70%	6,458,151	0.30%
21k_20x	219	3,403	2,111,409,317	99.70%	6,438,715	0.30%
21k_30x	314	3,322	2,119,300,293	99.70%	7,331,493	0.30%
21k_40x	335	3,134	2,117,617,369	99.50%	10,201,330	0.50%
21k_50x	353	3,234	2,117,547,664	99.40%	13,746,000	0.60%
21k_60x	347	3,138	2,119,811,928	99.30%	15,234,573	0.70%
21k_75x	347	3,137	2,115,452,448	99.10%	19,997,068	0.90%

Supplementary Table 5. BUSCO values of NC358 assemblies.

Assembly	Complete (%)	Duplicated* (%)	Fragmented (%)	Missing (%)
11k_50x_raw	92.2	3.7	2.4	5.4
16k_50x_raw	91.9	4.0	2.9	5.2
21k_20x_raw	52.6	1.8	7.2	40.2
21k_30x_raw	81.9	2.6	5.0	13.1
21k_40x_raw	88.4	3.2	3.8	7.8
21k_50x_raw	90.4	3.9	2.9	6.7
21k_60x_raw	89.8	3.9	3.9	6.3
21k_75x_raw	92.3	4.4	2.4	5.3
11k_50x_Pilon	95.7	5.2	1.0	3.3
16k_50x_Pilon	96.7	5.2	1.0	2.3
21k_20x_Pilon	68.0	2.4	3.1	28.9
21k_30x_Pilon	95.5	4.9	1.1	3.4
21k_40x_Pilon	96.5	5.1	1.1	2.4
21k_50x_Pilon	96.4	5.1	1.2	2.4
21k_60x_Pilon	96.2	5.1	1.4	2.4
21k_75x_Pilon	96.3	5.0	1.1	2.6

*Also included in the complete category.

Supplementary Table 6. Length distribution of gene features for NC358 21k_20x and 21k_75x*.

	21k_20x	21k_75x
gene count	28,275	39,578
gene length (mean)	5,139	4,789
gene length (mediar	2,878	2,977
exon count	159,748	225,849
exon length (mean)	269	277
exon length (mediar	145	147
intron count	131,473	186,271
intron length (mean	766	671
intron length (media	154	155
CDS count	35,452	50,044
CDS length (mean)	887	920
CDS length (mediar	867	918
peptide count	35,452	50,044
peptide length (mea	296	307
peptide length (med	289	306
exons per transcript	6	6
single-exon gene cc	5756 (20.4)	8211 (20.7)

*The longest CDS of a predicted protein locus was used to calculate CDS and protein length statistics

Supplementary Table 7. Cross checking *de-novo* gene predictions of the 21k_20x and 21k_75x assembly.

Assembly	Gene prediction	Gap (13N or 100N) containing genes	Uniq mapping	Multi mapping	No mapping	Translocation
11k_50x	NC358_21k_20x	137	25711 (90.93%)	2533 (8.96%)	50 (0.18%)	65 (0.23%)
16k_50x	NC358_21k_20x	35	25758 (91.1%)	2552 (9.03%)	0 (0%)	45 (0.16%)
21k_30x	NC358_21k_20x	51	25760 (91.11%)	2544 (9%)	6 (0.02%)	41 (0.15%)
21k_40x	NC358_21k_20x	25	25756 (91.09%)	2550 (9.02%)	3 (0.01%)	42 (0.15%)
21k_50x	NC358_21k_20x	15	25763 (91.12%)	2553 (9.03%)	1 (0%)	44 (0.16%)
21k_60x	NC358_21k_20x	12	25790 (91.21%)	2533 (8.96%)	5 (0.02%)	41 (0.15%)
21k_75x	NC358_21k_20x	12	25807 (91.27%)	2503 (8.85%)	19 (0.07%)	37 (0.13%)
11k_50x	NC358_21k_75x	163	36491 (92.2%)	2991 (7.56%)	108 (0.27%)	76 (0.19%)
16k_50x	NC358_21k_75x	47	36613 (92.51%)	3001 (7.58%)	10 (0.03%)	25 (0.06%)
21k_20x	NC358_21k_75x	604	31157 (78.72%)	4353 (11%)	3391 (8.57%)	1106 (2.79%)
21k_30x	NC358_21k_75x	107	36416 (92.01%)	3111 (7.86%)	52 (0.13%)	99 (0.25%)
21k_40x	NC358_21k_75x	46	36604 (92.49%)	3004 (7.59%)	11 (0.03%)	40 (0.1%)
21k_50x	NC358_21k_75x	33	36658 (92.62%)	2973 (7.51%)	4 (0.01%)	25 (0.06%)
21k_60x	NC358_21k_75x	23	36745 (92.84%)	2895 (7.31%)	4 (0.01%)	23 (0.06%)

Supplementary Table 8. Remapping of 6,428,286* unmapped reads from the 21k_20x assembly.

Assembly	% Uniquely mapped reads	% Multiple mapped reads	% Unmapped reads: too short	% Unmapped reads: other
11k_50x	35.58	0.48	58.32	5.55
16k_50x	36.43	0.57	57.37	5.52
21k_30x	35.40	0.47	58.45	5.62
21k_40x	36.32	0.41	57.57	5.63
21k_50x	36.34	0.44	57.52	5.64
21k_60x	36.43	0.44	57.41	5.63
21k_75x	36.25	0.45	57.61	5.63

*Derived from the 16DAP_embryo (MN22101) RNA-seq library.

Supplementary Table 9. Mapping unmapped RNA-seq reads from the 21k_20x assembly to the 21k_75x assembly.

Assembly	Tissue	Total remapped genes*	TE-contained	Full-length present in 21k_20x (All genes)**	Full-length present in 21k_20x (non-TE genes)
21k_75x	16DAP_embryo	1987	1306	259	35
21k_75x	16DAP_endosperm	1567	1006	187	35
21k_75x	8DAS_root	2091	1337	274	54
21k_75x	8DAS_shoot	1985	1295	244	34
21k_75x	R1_anther	1670	1056	174	27
21k_75x	V11_base	2251	1475	307	51
21k_75x	V11_middle	1868	1193	216	38
21k_75x	V11_tip	1648	1044	205	36
21k_75x	V18_ear	2097	1377	270	41
21k_75x	V18_tassel	2177	1425	272	41
All assemblies	All unique genes	3184	2144	640	136

*≥20% of the 21k_75x gene CDS was covered by 21k_20x-unmapped RNA-seq reads.

**≥80% of the 21k_75x remapped gene was aligned to the syntenic position in 21k_20x.

Supplementary Table 10. Assembly of *Rp1-D* and *zein* tandem gene arrays in NC358 assemblies.

Assembly	Locus	Chromosome	Start	End	Size	Gap	Bionano size	% assembled
11k_50x	*Rp1-D*	chr10	3208951	3744012	535,062	0	536,000	99.83%
16k_50x	*Rp1-D*	chr10	3220835	3756401	535,567	0	536,000	99.92%
21k_20x	*Rp1-D*	chr10	3174015	3659526	485,512	110,951	536,000	69.88%
21k_30x	*Rp1-D*	chr10	3230582	3764793	534,212	3,010	536,000	99.10%
21k_40x	*Rp1-D*	chr10	3243686	3778609	534,924	0	536,000	99.80%
21k_50x	*Rp1-D*	chr10	3220903	3756460	535,558	0	536,000	99.92%
21k_60x	*Rp1-D*	chr10	3222035	3757768	535,734	0	536,000	99.95%
21k_75x	*Rp1-D*	chr10	3222239	3757985	535,747	0	536,000	99.95%
11k_50x	*zein*	chr4	5235176	5297502	62,327	0	62,400	99.88%
16k_50x	*zein*	chr4	5067736	5130092	62,357	0	62,400	99.93%
21k_20x	*zein*	chr4	5094647	5151392	56,746	0	62,400	90.94%
21k_30x	*zein*	chr4	5227565	5289795	62,231	0	62,400	99.73%
21k_40x	*zein*	chr4	5153674	5215988	62,315	0	62,400	99.86%
21k_50x	*zein*	chr4	5238850	5301205	62,356	0	62,400	99.93%
21k_60x	*zein*	chr4	5267091	5329454	62,364	0	62,400	99.94%
21k_75x	*zein*	chr4	5251326	5313730	62,405	0	62,400	100.01%

Supplementary Table 11. Long terminal repeat (LTR) retrotransposon assembly.

Assembly	Chromosome	Start	End	Size	Ngap	Bionano size	Assembled (%)
11k_50x	8	18544831	18641401	96,571	10,194	96,000	89.98%
16k_50x	8	18404134	18500646	96,513	658	96,000	99.85%
21k_20x	8	18062087	18158365	96,279	26,866	96,000	72.31%
21k_30x	8	18320377	18416667	96,291	0	96,000	100.30%
21k_40x	8	18276855	18373357	96,503	0	96,000	100.52%
21k_50x	8	18291491	18388059	96,569	0	96,000	100.59%
21k_60x	8	18299388	18396000	96,613	0	96,000	100.64%
21k_75x	8	18279612	18375860	96,249	0	96,000	100.26%
11k_50x	7	10960484	11057176	96,693	0	97,000	99.68%
16k_50x	7	10819558	10916251	96,694	0	97,000	99.68%
21k_20x	7	10828869	10925502	96,634	0	97,000	99.62%
21k_30x	7	10815983	10912686	96,704	0	97,000	99.69%
21k_40x	7	10855912	10952671	96,760	0	97,000	99.75%
21k_50x	7	10846400	10943105	96,706	0	97,000	99.70%
21k_60x	7	10827931	10924717	96,787	0	97,000	99.78%
21k_75x	7	10828769	10925634	96,866	0	97,000	99.86%
11k_50x	4	69325213	69429905	104,693	0	98,500	106.29%
16k_50x	4	68762627	68861035	98,409	0	98,500	99.91%
21k_20x	4	68642261	68740649	98,389	0	98,500	99.89%
21k_30x	4	68722292	68820497	98,206	0	98,500	99.70%
21k_40x	4	68563105	68661473	98,369	0	98,500	99.87%
21k_50x	4	68825482	68923651	98,170	0	98,500	99.66%
21k_60x	4	67759980	67858288	98,309	0	98,500	99.81%
21k_75x	4	67913191	68011579	98,389	0	98,500	99.89%
11k_50x	8	117013605	117116490	102,886	0	102,000	100.87%
16k_50x	8	115714149	115817084	102,936	0	102,000	100.92%
21k_20x	8	115147939	115280559	132,621	0	102,000	130.02%
21k_30x	8	115457898	115560587	102,690	0	102,000	100.68%
21k_40x	8	115108542	115211271	102,730	0	102,000	100.72%
21k_50x	8	115084159	115187022	102,864	0	102,000	100.85%
21k_60x	8	115383730	115486308	102,579	0	102,000	100.57%
21k_75x	8	109748679	109851210	102,532	0	102,000	100.52%
11k_50x	4	189900740	190019386	118,647	0	119,000	99.70%
16k_50x	4	187655422	187774126	118,705	0	119,000	99.75%
21k_20x	4	187052370	187171141	118,772	0	119,000	99.81%
21k_30x	4	187723697	187842130	118,434	0	119,000	99.52%
21k_40x	4	187217758	187336374	118,617	0	119,000	99.68%
21k_50x	4	187024271	187142973	118,703	0	119,000	99.75%
21k_60x	4	186488924	186607568	118,645	0	119,000	99.70%
21k_75x	4	186500813	186619584	118,772	0	119,000	99.81%

Supplementary Table 12. Assembly of the bz locus in NC358 assembl

Assembly	Chromosome	Start	End	Assembled size	Gap	Bionano size	% assembled
11k_50x	9	11,645,901	11949398	303498	0	303500	100.00%
16k_50x	9	11645817	11934876	289060	0	303500	95.24%
21k_20x	9	11609450	11914852	305403	134664	303500	56.26%
21k_30x	9	11450197	11758547	308351	0	303500	101.60%
21k_40x	9	11684275	11995285	311011	0	303500	102.47%
21k_50x	9	11710831	11999843	289013	0	303500	95.23%
21k_60x	9	11645306	11934397	289092	0	303500	95.25%
21k_75x	9	11625031	11914133	289103	0	303500	95.26%
canu_only	9	11618783	11908063	289281	0	303500	95.31%
falcon_only	9	11613572	11919956	306385	0	303500	100.95%

Supplementary Table 13. Terminations and characteristics of genomic components used in this study.

Mesurement	Features	Typical size range	Explanations
BUSCO	Gene	<10 kb	Universal single-copy orthologs
LAI	LTR retrotransposons	~10 kb	Estimate the assembly quality of intact LTR retrotransposons
LTR	LTR retrotransposons	20-120 kb	Clusters of LTR retrotransposons
bz locus	LTR retrotransposons	200-300 kb	The bz gene locus enriched with retrotransposons
Tandem genes	Gene array	50-500 kb	Clusters of tandem duplicated genes
Telomere	TTTAGGG and CCCTAAA arrays	0 - 500 kb	Clusters of telomeric 7-mer arrays
Subtelomere	Tandem repeat	0 - 650 kb	Clusters of 300 - 1300 bp tandem repeats near telomere regions
CentC	CentC	200 kb - 1.1 Mb	Clusters of the 156 bp CentC arrays
Knob	knob180 and TR-1	2-3 Mb	Clusters of the 180 bp knob and 350 bp TR-1 tandem arrays
NOR	rDNA	~9 Mb	A cluster of ribosomal DNA sequences (~11 kb) on chromosome 6

Supplementary Table 14. Telomere 7-mer count in NC358 assemblies.

Assembly	All	chr1	chr2	chr3	chr4	chr5	chr6	chr7	chr8	chr9	chr10
11k_50x	706	37	15	115	57	0	0	355	44	83	0
16k_50x	773	41	16	117	101	14	0	301	57	0	126
21k_20x	125	7	0	37	33	0	0	34	11	0	3
21k_30x	515	0	20	45	70	1	0	319	1	32	27
21k_40x	696	0	18	19	44	15	0	348	84	95	73
21k_50x	1488	47	20	139	252	15	0	502	211	97	205
21k_60x	1839	59	15	148	488	11	0	582	84	210	242
21k_75x	2024	64	17	131	583	13	0	452	320	227	217

Supplementary Table 15. Assembly of telomere-subtelomere regions in the NC358 21k_75x assembly.

Assembly	Location	Chromosome	Telomere start	Telomere end	Telomere assembly length	Telomere 7-mer count	Telomere Ngap	Subtelomere start	Subtelomere end	Subtelomere assembly length	Subtelomere Ngap	Subtelomere 7-mer count	Telomere-Subtelomere Bionano size	Telomere-Subtelomere assembled (%)
21k_75x	Head	1	1	433468	433,468	0	0	433469	435337	1,869	0	31	577,000	75.45%
21k_75x	Head	2	1	394247	394,247	0	0	394248	445868	51,621	0	30	462,000	96.51%
21k_75x	Head	3	1	47069	47,069	115	0	47070	73320	26,251	0	460	75,000	97.76%
21k_75x	Head	4	1	2982	2,982	269	0	2983	272759	269,777	0	975	279,000	97.76%
21k_75x	Head	5	1	154	154	2	0	155	18963	18,809	0	62	450,000	4.21%
21k_75x	Head	6	NA	NA	NA	NA	NA	NA	NA	NA	NA	NA	375,000	0.00%
21k_75x	Head	7	1	29757	29,757	204	0	NA	NA	NA	NA	NA	29,000	102.61%
21k_75x	Head	8	1	22432	22,432	31	0	NA	NA	NA	NA	NA	26,000	86.28%
21k_75x	Head	9	NA	NA	NA	NA	NA	NA	NA	NA	NA	NA	50,000	0.00%
21k_75x	Head	10	1	965	965	61	0	NA	NA	NA	NA	NA	3,000	32.17%
21k_75x	Tail	1	305252324	305252879	556	64	0	305238204	305252323	14,120	0	228	16,000	91.73%
21k_75x	Tail	2	244815481	244815626	146	17	0	244691296	244815480	124,185	0	264	119,000	104.48%
21k_75x	Tail	3	237295292	237295441	150	16	0	237259706	237295291	35,586	0	261	128,000	27.92%
21k_75x	Tail	4	254016477	254019491	3,015	314	0	253956476	254016476	60,001	0	495	67,000	94.05%
21k_75x	Tail	5	221818660	221818757	98	11	0	221698192	221818659	120,468	0	532	122,000	98.82%
21k_75x	Tail	6	177187343	177192286	4,944	0	0	176647461	177187342	539,882	0	20	615,000	88.59%
21k_75x	Tail	7	181303722	181309976	6,255	248	0	181303542	181303721	180	0	248	8,000	80.44%
21k_75x	Tail	8	173323222	173326872	3,651	289	0	173238252	173323221	84,970	0	499	87,000	101.86%
21k_75x	Tail	9	165074204	165125378	51,175	227	0	165067267	165074203	6,937	0	231	60,000	96.85%
21k_75x	Tail	10	155293247	155297942	4,696	156	0	155217671	155293246	75,576	0	170	81,000	99.10%

Supplementary Table 16. Assembly of CentC arrays in NC358 assemblies.

Assembly	Chromosome	Start	End	Size	CentC	Ngap	Bionano size	Assembled (%)
11k_50x	1	137484000	137542000	58000	28015	0	1143000	5.07%
16k_50x	1	135639000	136248000	609000	210413	100	1143000	53.27%
21k_20x	1	135864000	135991000	127000	39126	100	1143000	11.10%
21k_30x	1	135514000	135871000	357000	87381	100	1143000	31.22%
21k_40x	1	135375000	135899000	524000	152651	10942	1143000	44.89%
21k_50x	1	135408000	136308000	900000	374351	100	1143000	78.73%
21k_60x	1	135267000	136335000	1068000	467666	100	1143000	93.43%
21k_75x	1	135546000	136707000	1161000	530143	100	1143000	101.57%
11k_50x	2	98541000	98825000	284000	115533	40385	988000	24.66%
16k_50x	2	97769000	98181000	412000	191312	100	988000	41.69%
21k_20x	2	96678000	96703000	25000	7819	100	988000	2.52%
21k_30x	2	97375000	97423000	48000	16513	100	988000	4.85%
21k_40x	2	97313000	97316000	3000	1117	0	988000	0.30%
21k_50x	2	97193000	97398000	205000	86772	100	988000	20.74%
21k_60x	2	97255000	97571000	316000	174399	100	988000	31.97%
21k_75x	2	97118000	97419000	301000	160498	100	988000	30.46%
11k_50x	3	88912000	89076000	164000	55510	100	577000	28.41%
16k_50x	3	87724000	88175000	451000	183278	0	577000	78.16%
21k_20x	3	87644000	87663000	19000	11108	0	577000	3.29%
21k_30x	3	87727000	88082000	355000	159098	100	577000	61.51%
21k_40x	3	87445000	87895000	450000	183193	0	577000	77.99%
21k_50x	3	87462000	87909000	447000	180122	0	577000	77.47%
21k_60x	3	87471000	87922000	451000	183319	0	577000	78.16%
21k_75x	3	87458000	87907000	449000	181527	0	577000	77.82%
11k_50x	4	112106000	112354000	248000	23214	0	208000	119.23%
16k_50x	4	110896000	111026000	130000	16981	0	208000	62.50%
21k_20x	4	110641000	110795000	154000	9996	0	208000	74.04%
21k_30x	4	111017000	111223000	206000	18033	0	208000	99.04%
21k_40x	4	110947000	111154000	207000	18007	0	208000	99.52%
21k_50x	4	110981000	111188000	207000	18235	0	208000	99.52%
21k_60x	4	110021000	110228000	207000	18337	0	208000	99.52%
21k_75x	4	110091000	110298000	207000	18211	0	208000	99.52%
11k_50x	5	107953000	108172000	219000	78095	18086	269000	74.69%
16k_50x	5	106322000	106541000	219000	89732	0	269000	81.41%
21k_20x	5	106280000	106292000	12000	5819	0	269000	4.46%
21k_30x	5	106572000	106790000	218000	84815	4311	269000	79.44%
21k_40x	5	106285000	106504000	219000	89601	0	269000	81.41%
21k_50x	5	106179000	106398000	219000	89163	0	269000	81.41%
21k_60x	5	106397000	106616000	219000	89633	0	269000	81.41%
21k_75x	5	106497000	106740000	243000	101634	0	269000	90.33%
11k_50x	6	52985000	53313000	328000	100706	0	245000	133.88%
16k_50x	6	53049000	53511000	462000	190943	100	245000	188.53%
21k_20x	6	NA	NA	NA	NA	NA	245000	0.00%
21k_30x	6	53508000	53596000	88000	37692	100	245000	35.88%
21k_40x	6	52688000	53180000	492000	206531	100	245000	200.78%
21k_50x	6	53487000	53961000	474000	195659	100	245000	193.43%
21k_60x	6	53130000	53846000	716000	347053	100	245000	292.20%
21k_75x	6	54010000	54740000	730000	365362	100	245000	297.92%
11k_50x	7	58388000	58680000	292000	30441	178500	294000	38.61%
16k_50x	7	57788000	58080000	292000	90462	113686	294000	60.65%
21k_20x	7	57652000	57791000	139000	20095	56075	294000	28.21%
21k_30x	7	57762000	58054000	292000	58983	144652	294000	50.12%
21k_40x	7	57740000	58033000	293000	110789	85106	294000	70.71%
21k_50x	7	57793000	58085000	292000	115991	80079	294000	72.08%
21k_60x	7	57762000	58067000	305000	137849	60257	294000	83.25%
21k_75x	7	57788000	58081000	293000	162015	33262	294000	88.35%
11k_50x	8	49412000	49425000	13000	5268	0	332000	3.92%
16k_50x	8	48977000	48999000	22000	14154	100	332000	6.60%
21k_20x	8	48450000	48458000	8000	1056	100	332000	2.38%
21k_30x	8	48713000	48736000	23000	14716	100	332000	6.90%
21k_40x	8	48761000	48791000	30000	21606	100	332000	9.01%
21k_50x	8	48793000	48840000	47000	38096	100	332000	14.13%
21k_60x	8	48802000	49067000	265000	136180	100	332000	79.79%
21k_75x	8	48820000	49321000	501000	259956	100	332000	150.87%
11k_50x	9	59744000	60299000	555000	49289	238637	889000	35.59%
16k_50x	9	59375000	59819000	444000	51180	222726	889000	24.89%
21k_20x	9	59140000	59435000	295000	13033	248205	889000	5.26%
21k_30x	9	59106000	59631000	525000	54165	231938	889000	32.97%
21k_40x	9	59174000	59698000	524000	46880	237534	889000	32.22%
21k_50x	9	59020000	59647000	627000	185642	31801	889000	66.95%
21k_60x	9	59053000	60086000	1033000	263496	222791	889000	91.14%
21k_75x	9	59201000	60232000	1031000	339464	94154	889000	105.38%
11k_50x	10	54103000	54326000	223000	102328	26037	770000	25.58%
16k_50x	10	53611000	53941000	330000	191572	100	770000	42.84%
21k_20x	10	NA	NA	NA	NA	NA	770000	0.00%
21k_30x	10	53684000	53921000	237000	137381	3989	770000	30.26%
21k_40x	10	53372000	53708000	336000	182180	4885	770000	43.00%
21k_50x	10	53362000	53633000	271000	147176	100	770000	35.18%
21k_60x	10	53431000	53772000	341000	203121	100	770000	44.27%
21k_75x	10	53360000	53650000	290000	164608	0	770000	37.66%

Supplementary Table 17. Assembly of the nucleolus organizer region (NOR) in NC358 assemblies.

Assembly	Chromosome	Start	End	Size	Ngap	Bionano size	Assembled (%)
11k_50x	6	11655000	17858000	6203000	1064982	8963000	57.32%
16k_50x	6	11585000	18237000	6652000	590664	8963000	67.63%
21k_20x	6	11767000	18587000	6820000	2185636	8963000	51.71%
21k_30x	6	11907000	18898000	6991000	575011	8963000	71.58%
21k_40x	6	11527000	18159000	6632000	154723	8963000	72.27%
21k_50x	6	11963000	18938000	6975000	150001	8963000	76.15%
21k_60x	6	11525000	18550000	7025000	163503	8963000	76.55%
21k_75x	6	11548000	19411000	7863000	593178	8963000	81.11%

Supplementary Table 18. Assembly of two knobs in NC358 assemblies.

Assembly	Chromosome	Start	End	Size	Knob180	TR-1	Ngap	Bionano size	Assembled (%)
11k_50x	7	155075000	155132000	57000	23231	0	100	2384000	2.39%
16k_50x	7	153196000	153598000	402000	79037	10178	255925	2384000	6.13%
21k_20x	7	152633000	152684000	51000	21256	0	100	2384000	2.14%
21k_30x	7	153070000	154945000	1875000	339637	7895	1291298	2384000	24.48%
21k_40x	7	152935000	154786000	1851000	513859	18035	1061083	2384000	33.13%
21k_50x	7	152912000	154936000	2024000	788186	38321	806153	2384000	51.08%
21k_60x	7	152908000	155188000	2280000	1194099	68683	549710	2384000	72.58%
21k_75x	7	153072000	155113000	2041000	1221516	40958	260205	2384000	74.70%
11k_50x	4	221045000	222348000	1303000	157762	30155	901518	2716000	14.78%
16k_50x	4	218442000	219763000	1321000	288592	41041	710991	2716000	22.46%
21k_20x	4	217910000	218573000	663000	88974	30075	383515	2716000	10.29%
21k_30x	4	218528000	219310000	782000	209568	40875	333034	2716000	16.53%
21k_40x	4	218054000	219377000	1323000	464723	59296	433929	2716000	32.73%
21k_50x	4	217795000	218909000	1114000	457817	59406	119473	2716000	36.62%
21k_60x	4	217285000	219097000	1812000	800467	95167	458797	2716000	49.82%
21k_75x	4	217256000	219078000	1822000	963576	95222	269819	2716000	57.15%

Supplementary Table 19. Mapping NC358 RNA-seq libraries to the B73v4 genome.

Sample	ID	TotalReads	TotalAvgLen	TotalMapped	TotalMapped%	UniqMapped%	MultiMapped%	Coverage
16DAP_embryo	MN22101	26,676,494	151	23,561,411	88.32	83.62	4.70	23.83
16DAP_embryo	MN22102	21,849,863	151	19,942,677	91.27	86.85	4.42	19.52
16DAP_endosperm	MN22091	19,411,672	151	16,884,165	86.98	73.35	13.63	17.34
16DAP_endosperm	MN22092	25,886,468	151	19,628,392	75.82	61.71	14.12	23.13
8DAS_root	MN22011	29,944,693	151	27,412,746	91.54	87.08	4.46	26.75
8DAS_root	MN22012	25,302,835	151	22,911,846	90.55	86.15	4.41	22.60
8DAS_shoot	MN22021	25,436,184	151	21,799,056	85.70	79.39	6.31	22.72
8DAS_shoot	MN22022	26,195,884	151	23,898,108	91.23	85.38	5.85	23.40
R1_anther	MN22081	19,825,786	151	16,575,491	83.61	77.88	5.73	17.71
R1_anther	MN22082	16,061,615	151	13,277,128	82.66	76.84	5.82	14.35
V11_base	MN22031	28,490,315	151	25,221,965	88.53	83.79	4.74	25.45
V11_base	MN22032	23,021,823	151	20,614,586	89.54	84.84	4.70	20.57
V11_middle	MN22041	22,071,843	151	17,915,144	81.17	74.84	6.33	19.72
V11_middle	MN22042	21,159,572	151	17,092,816	80.78	74.62	6.16	18.90
V11_tip	MN22051	28,907,122	151	23,990,616	82.99	75.55	7.44	25.82
V11_tip	MN22052	23,156,765	151	18,786,716	81.13	73.81	7.31	20.69
V18_ear	MN22071	28,053,505	151	25,725,011	91.70	87.31	4.39	25.06
V18_ear	MN22073	25,797,093	151	23,370,930	90.60	86.18	4.41	23.05
V18_tassel	MN22061	24,269,086	151	21,208,234	87.39	82.10	5.29	21.68
V18_tassel	MN22062	27,868,407	151	24,270,956	87.09	81.68	5.41	24.90
