# Supplementary material for: Effect of sequence depth and length in long-read assembly of the maize inbred NC358
Source: Nat Commun. 2020 May 8;11:2288. doi: 10.1038/s41467-020-16037-7 (PMC7211024; doi:10.1038/s41467-020-16037-7)
Supplement: Supplementary file 3 — Reporting Summary [file 41467_2020_16037_MOESM3_ESM.pdf]

nature research

Corresponding author(s): R. Kelly Dawe, Candice N. Hirsch, Matthew B. Hufford, Doreen Ware

Last updated by author(s): Mar 10, 2020

Reporting Summary

Nature Research wishes to improve the reproducibility of the work that we publish. This form provides structure for consistency and transparency in reporting. For further information on Nature Research policies, see Authors & Referees and the Editorial Policy Checklist .

Statistics

For all statistical analyses, confirm that the following items are present in the figure legend, table legend, main text, or Methods section.

n/a	Confirmed	
x		The exact sample size (*n*) for each experimental group/condition, given as a discrete number and unit of measurement
	x	A statement on whether measurements were taken from distinct samples or whether the same sample was measured repeatedly
	x	The statistical test(s) used AND whether they are one- or two-sided<br>*Only common tests should be described solely by name; describe more complex techniques in the Methods section.*
x		A description of all covariates tested
	x	A description of any assumptions or corrections, such as tests of normality and adjustment for multiple comparisons
	x	A full description of the statistical parameters including central tendency (e.g. means) or other basic estimates (e.g. regression coefficient) AND variation (e.g. standard deviation) or associated estimates of uncertainty (e.g. confidence intervals)
	x	For null hypothesis testing, the test statistic (e.g. *F*, *t*, *r*) with confidence intervals, effect sizes, degrees of freedom and *P* value noted<br>*Give P values as exact values whenever suitable.*
x		For Bayesian analysis, information on the choice of priors and Markov chain Monte Carlo settings
x		For hierarchical and complex designs, identification of the appropriate level for tests and full reporting of outcomes
x		Estimates of effect sizes (e.g. Cohen's *d*, Pearson's *r*), indicating how they were calculated

Our web collection on statistics for biologists contains articles on many of the points above.

Software and code

Policy information about availability of computer code

Data collection	Bionano Saphyr Compute (80013), Bionano Compute Server (80014)
Data analysis	SequelQC (dd0f735), BaseSpase (v5.28), seqtk (v1.2), falcon_kit (v0.7), Canu (v1.7), R (v3.5.1), GenomeQC (8f60d17), BUSCO (v3.0.2), BLAST (v2.6), HMMER (v3.1b2), Augustus (v3.3), LTR_retriever (v2.6), LAI (vbeta3.2), BLASR (5afe2f9), SMRT Link (v5.1.0), Minimap2 (v2.16), Pilon (v1.23-0), Bionano Solve (v3.2.1_04122018), BionanoSolve (v3.4), ALLMAPS (v0.8.12), HiSat2 (v2.1.0), RepeatMasker (v4.0.9), Minimap2 (v2.2), dotPlotly (10/29/17), MAKER-P, FGENESH, STAR (v2.5.2b), SAMtools (v1.9), BEDtools (v2.28.0), TEsorter (v1.1.4), EMBOSS (v6.6.0), RepeatMasker (v4.0.7), Jellyfish (v2.0), Fragment Analyzer, Flye (v2.6), Peregrine (pg0.1.6.1), WTDBG2 (v2.5)<br>All code developed for this study is available on GitHub: https://github.com/HuffordLab/Maize_NC358.

For manuscripts utilizing custom algorithms or software that are central to the research but not yet described in published literature, software must be made available to editors/reviewers. We strongly encourage code deposition in a community repository (e.g. GitHub). See the Nature Research guidelines for submitting code & software for further information.

Data

Policy information about availability of data

All manuscripts must include a data availability statement. This statement should provide the following information, where applicable:

- Accession codes, unique identifiers, or web links for publicly available datasets
- A list of figures that have associated raw data
- A description of any restrictions on data availability

PacBio and Illumina sequencing reads for the NC358 line used in this study are available with EBI Biosample ID ERS3120561 [https://www.ebi.ac.uk/ena/data/view/ERS3120561]. PacBio SMRT subreads for the maize inbred line B73 (sequenced to 68x depth) were retrieved from the NCBI SRA database with accession ID SRX1472849 [https://www.ncbi.nlm.nih.gov/sra/SRX1472849]. PacBio SMRT subreads for the human HG002 sample (sequenced to 147x depth) were retrieved with accession IDs SRX1033793 and SRX1033794 [https://www.ncbi.nlm.nih.gov/sra/SRX1033794]. The source data underlying Figs 1BCFG, 2ABI, Supplementary Figs 2, 4, 6-11, 15-16, and Supplementary Table 3 are provided as a Source Data file. Data supporting the findings of this work are available within the paper and its

Supplementary Information files. A reporting summary for this Article is available as a Supplementary Information file. The datasets generated and analyzed during the current study are available from the corresponding author upon request. All code developed for this study is available on GitHub: https://github.com/HuffordLab/Maize_NC358.

Field-specific reporting

Please select the one below that is the best fit for your research. If you are not sure, read the appropriate sections before making your selection.

- [x] Life sciences
- [ ] Behavioural & social sciences
- [ ] Ecological, evolutionary & environmental sciences

For a reference copy of the document with all sections, see nature.com/documents/nr-reporting-summary-flat.pdf

Life sciences study design

All studies must disclose on these points even when the disclosure is negative.

Sample size	The depth of sequence necessary for genome assembly in maize was informed by previously published long-read assemblies within the species (Jiao et al. 2017, PMID: 28605751, sequenced the maize inbred line B73 for 68x) and by our own experiments with the assembly pipeline.
Data exclusions	Our full data set for genome assembly was subsampled at varying read depth and read length to evaluate the impact on assembly contiguity and completeness. Subreads were randomly subsampled and the unsampled reads were excluded in the subsamples.
Replication	Assembly is highly computationally intensive and results should not vary considerably across runs, so we did not replicate assembly at any given read depth or read length.
Randomization	Reads were randomly subsampled for assembly, but were trimmed to approximate the read length distribution observed in typical PacBio SMRT cell runs.
Blinding	Blinding is not necessary for running assembly algorithms or for characterizing their output.

Reporting for specific materials, systems and methods

We require information from authors about some types of materials, experimental systems and methods used in many studies. Here, indicate whether each material, system or method listed is relevant to your study. If you are not sure if a list item applies to your research, read the appropriate section before selecting a response.

Materials & experimental systems

n/a	Involved in the study
[x]	[ ] Antibodies
[x]	[ ] Eukaryotic cell lines
[x]	[ ] Palaeontology
[x]	[ ] Animals and other organisms
[x]	[ ] Human research participants
[x]	[ ] Clinical data

Methods

n/a	Involved in the study
[x]	[ ] ChIP-seq
[x]	[ ] Flow cytometry
[x]	[ ] MRI-based neuroimaging
