# Supplementary material for: Effect of sequence depth and length in long-read assembly of the maize inbred NC358
Source: Nat Commun. 2020 May 8;11:2288. doi: 10.1038/s41467-020-16037-7 (PMC7211024; doi:10.1038/s41467-020-16037-7)
Supplement: Supplementary file 2 — Peer Review [file 41467_2020_16037_MOESM2_ESM.pdf]

Editorial Note: Parts of this peer review file have been redacted as indicated to remove third-party material where no permission to publish could be obtained.

Reviewers' comments:

Reviewer #1 (Remarks to the Author):

In the manuscript entitled “Effect of Sequence Depth and Length in Long-read Assembly of the Maize Inbred NC358,” Ou et al., leverage a high-quality set of Pacific Bioscience reads to develop guidelines for sequencing and assembly for a complex plant genome. The authors generate a high coverage, long read dataset, and analyze eight different assemblies varying read depth and read length N50 with three different assembly approaches. They scaffold the assemblies using BioNano optical maps and anchor into chromosomes using GoldenGate and pan-genome genetic markers. Then the authors carry out some very nice comparisons of the genome assemblies looking at completeness (BUSCO), complex repeats (centromeres, rDNA, etc.) and transposable elements. The authors argue, and not surprisingly, that higher coverage and longer reads generate higher quality assemblies. Specifically, they show for a plant that has a predominant 10kb repeat structure, reads exceeding that repeat length and coverage enable assembly of more of the overall repeat content. Specifically, the use of BioNano maps to validate the assembly contiguity and repeat content is interesting and compelling. Overall, the concept of this manuscript is on point and useful to the genome assembly and plant communities. However, the separation in assemblies could be broader, more assembly methods could be tested, and the results need to be placed more clearly in the current state of the art in plant genome assembly.

The introduction is missing some key background to set the stage for this work. First off, the authors do not introduce the current state genome assembly using long reads in terms of platforms, tools and results. Multiple papers have addressed the different long read assemblers and approaches; alerting the reader to this background will greatly improve the introduction. There are many genomes published using long reads at this point, some that are extremely high quality such the telomere to telomere rice genome, and it would put the current work into context based on these previous results. Moreover, background on the Maize genome and its complexity would also place the current work in context. The last author published the B73v4 PacBio assembly and some introduction to this work would allow the reader to understand the complexity of the Maize genome. The authors compare the Arabidopsis and wheat genomes; this comparison is less relevant to the current work since the Maize genome is diploid and complex in a different way than wheat.

In terms of methodology it would greatly increase the power of this analysis if the authors extend their analysis to different long read assemblers, and mixes of data that create greater difference in the resulting assemblies. The authors focus on Canu and Falcon as well as a hybrid assembly method (really just a difference in error correction), but they don’t test several other popular long read assemblers like FlyE, WTDBG2, MECAT2 and minimap2/miniasm. Also, the authors only tested assemblies post error correction. The other long read assemblers mentioned above also work on uncorrected read, which would be a very interesting and important comparison since error correction consumes a lot of compute time. Recently the developer of Falcon released a new assembler called Peregrine based on minimizers of minimizers that works on high-quality PacBio reads (really meant for HiFi circular consensus reads but may work on the reads presented here). It would be extremely interesting to see how Peregrine performs on this Maize dataset; if not then a mention in the introduction or discussion concerning future developments. Since much has been done to compare many of these tools both in the literature and online across several species, the authors really need to place their findings squarely in the context of how this helps plant biologists beyond what is already known.

Two things in the text that were not clear and require some mention. First, the authors refer to the reads as "raw reads" in the text. Are these raw polymerase reads or subreads? In the methods it seems the authors are using "raw reads" in the body to mean subreads. This needs to be clear in the body. Second the authors mention the B73v4 and human PacBio datasets, and in there are some mention of them in the methods as well as supplement. The relevant data is in Table S2, and this is referenced once at line 72. Currently the relevance of adding this dataset is not clear in the body of the text; a sentence to highlight the comparison in Table S2 could make this clearer.

Minor points
Line 96: should it read dramatic read in contiguity since the reference is to contig N50?

Figure 1I: It is difficult to determine the number of SVs from the graph; would be nice to get a sense of number as well as size.

Line133: Why only annotate two assemblies? Where the missing genes found in the gaps paralogs?

At what level are the heuristics of the assemblers impacting the results? For instance, is there a coverage threshold that makes the 20x coverage assembly break at some points? Since the authors of Canu are on the manuscript some mention of assembler strengths and limitations would be informative.

If other assemblers were/are not tested, a justification of why only these long read assemblers were used would be good for the reader.

Reviewer #2 (Remarks to the Author):

This paper gives a detailed report of an in-depth evaluation on how to use long-read sequencing data to produce a high-quality assembly for a complex plant genome. Focusing on maize, the authors report assembly results using differently sized data sets and make a completeness assessment of genes, transposable elements, and highly repetitive features. Overall, the applied strategy and methods are sound but on some points, for example when discussing genic regions, the reported results are incomplete and as such do not offer detailed information on potential consequences for downstream biological data analysis (which is of major importance for most plant biologists). Whereas most of the results are in line with previous reports on long-read sequencing, this paper does describe a useful resource reference to the community.

Major
1. The title and abstract are somewhat misleading, as all evaluated assemblies (Table 1) are not the result of solely assembling an increasing number of long reads sequences, they also contain information from optical maps and GoldenGate genetic markers to perform superscaffolding. As such, most of the reported results do not only deal with long read assembly, but include additional assembly strategies which also come at a cost. Related to terminology, if in the text the term superscaffolding is used, please also use this term in Table 1 (as from your text I conclude you report N50 stats on superscaffolds here).
2. I am missing some key references which should be integrated to put the presented results in context. For example, the Belser et al paper (https://www.nature.com/articles/s41477-018-0289-4) generated and evaluated genome assembly using nanopore long reads for 3 different plant species. Likewise, Van Bel et al (https://www.ncbi.nlm.nih.gov/pubmed/30797187) gave a detailed overview of different data types and methods to assess completeness and actually performed a gene space comparison on different maize B73 assemblies. None of the presented results are discussed in light of these papers or other literature, which is extensive, on genome assembly strategies for complex plant/eukaryotic genomes.
3. The evaluation of the genic regions (line 133-) reports some mapping statistics but lacks strong

conclusions. Based on Figure 1G and Table S17, I learned that the RNA-Seq mapping rate for the NC358 tissue samples is higher for the B73 genome compared to the best NC358 assembly. This indicates some of the maize gene space present in B73 and NC358 is missing in the NC358 assemblies, though this is not discussed. Are these repeat-rich genes like disease resistant genes or are these genes located near centro/telomeres? It would be good to also differentiate between multimapped and non-mapping RNA-Seq reads on the NC358 assemblies. It would be informative of the authors can report how many and which type of genes these are missing in the NC358 assemblies, based on the B73 gene models recovered by the NC358 RNA-Seq data, as this has major consequences for downstream gene-based anaslyses. Furthermore, taking the B73 gene models as a reference (though the Van Bel paper indicates >1000 of full-length genes present in V3 were removed in V4, which came as a surprise to me), how many of these genes are found back as full-length/partial, models in the NC358 assemblies? This would help to put the "obtained a total of 28,275 and 39,578 genes" (line 134) in perspective and could reveal if the best N50/contiguity assemblies also correspond with the assemblies best representing the maize gene space.

Minor
4. For the downsampling to lower coverages, every time only one sub-sample was selected, assembled and analyzed. Although this reviewer acknowledges that generating assemblies is resource intensive, it would be good to see, for one/few of the lower coverages, how big the standard deviations are on the reported statistics in Table 1, when considering multiple sub-samples (e.g. n=5).
5. If the aim of this paper is serving as a guide for future plant sequencing initiatives, adding a brief analysis of the costs associated with the different data types could be relevant.

Reviewer #3 (Remarks to the Author):

This manuscript focuses on practical, technical concerns for the many biological researchers who are using next generation genomic sequencing technologies to advance their research interests. Getting accurate and comprehensive sequencing results from plant genomes is especially difficult. In this set of studies, the authors have compared different datasets for a strain of maize to help determine what characters are most important in providing good, useful assembled sequences according to different criteria. PacBio and Illumina sequencing platforms were used to provide hybrid assemblies of excellent quality. Further, they use BioNano chromosomal mapping to extend their results. By comparing the use of different PacBio read lengths, read-depths, and evaluating each for different applications, the authors provide very useful information for others seeking to make best use of their resources for accomplishing their individual goals. The presentation is well-written, clear, and effective in using graphs. The research is very detailed, even exhaustive and should be a great example for others wanting to take on the daunting task of sequencing a genome as difficult as many plant genomes are (including maize). The only question I have, and it is only an academic one is whether they had thoughts or experience with respect to Oxford Nanopore technology, and whether their would be a value of including this platform for sequencing in cases where it seems (according to the current study) that read length seems most important to the quality of the results. The authors can address this if they wish. I think the manuscript should be accepted for publication. I came across no errors that needed to be corrected at this time.

Dear Editor and Reviewers,

We appreciate the careful reading of our manuscript and the constructive comments we have received. We have addressed concerns in our response below and in a revised version of our manuscript and think that this has substantially improved the paper. Our responses are presented in the non-italic text below.

Reviewers' comments:

Reviewer #1 (Remarks to the Author):

In the manuscript entitled "Effect of Sequence Depth and Length in Long-read Assembly of the Maize Inbred NC358," Ou et al., leverage a high-quality set of Pacific Bioscience reads to develop guidelines for sequencing and assembly for a complex plant genome. The authors generate a high coverage, long read dataset, and analyze eight different assemblies varying read depth and read length N50 with three different assembly approaches. They scaffold the assemblies using BioNano optical maps and anchor into chromosomes using GoldenGate and pan-genome genetic markers. Then the authors carry out some very nice comparisons of the genome assemblies looking at completeness (BUSCO), complex repeats (centromeres, rDNA, etc.) and transposable elements. The authors argue, and not surprisingly, that higher coverage and longer reads generate higher quality assemblies. Specifically, they show for a plant that has a predominant 10kb repeat structure, reads exceeding that repeat length and coverage enable assembly of more of the overall repeat content. Specifically, the use of BioNano maps to validate the assembly contiguity and repeat content is interesting and compelling. Overall, the concept of this manuscript is on point and useful to the genome assembly and plant communities. However, the separation in assemblies could be broader, more assembly methods could be tested, and the results need to be placed more clearly in the current state of the art in plant genome assembly.

Response:

Thank you for this nice summary of our study and we agree that the read length variation and use of Bionano maps are two key contributions of the work. We provide point-by-point responses for additional comments below.

=======

1. The introduction is missing some key background to set the stage for this work. First off, the authors do not introduce the current state genome assembly using long reads in terms of platforms, tools and results. Multiple papers have addressed the different long read assemblers and approaches; alerting the reader to this background will greatly improve the introduction. There are many genomes published using long reads at this point, some that are extremely high quality such the telomere to telomere rice genome, and it would put the current work into context based on these previous results. Moreover, background on the Maize genome and its complexity would also place the current work in context. The last author published the B73v4 PacBio assembly and some introduction to this work would allow the reader to understand the

complexity of the Maize genome. The authors compare the Arabidopsis and wheat genomes; this comparison is less relevant to the current work since the Maize genome is diploid and complex in a different way than wheat.

Response:

Thank you for your constructive suggestions. We have rewritten the introduction to include the current state of long-read genome assembly. A number of studies comparing different long-read assemblers were added to the background of this study. Some of the high-quality genome assemblies were also mentioned. We have removed the wheat genome comparison and instead added more context regarding the complexity of maize genomes and the current state of its sequencing efforts.

==========

2. In terms of methodology it would greatly increase the power of this analysis if the authors extend their analysis to different long read assemblers, and mixes of data that create greater difference in the resulting assemblies. The authors focus on Canu and Falcon as well as a hybrid assembly method (really just a difference in error correction), but they don't test several other popular long read assemblers like FlyE, WTDBG2, MECAT2 and minimap2/miniasm. Also, the authors only tested assemblies post error correction. The other long read assemblers mentioned above also work on uncorrected read, which would be a very interesting and important comparison since error correction consumes a lot of compute time.

Response:

The goal of this study is to explore the impact of different read lengths and coverage in the assembly of repetitive regions rather than a deep comparison of all available long-read assemblers. We initially tested Falcon and Canu because they are the most widely used assembly tools to create high-quality genome assembly and they are Overlap Layout Consensus (OLC)-based tools that can utilize long reads to create a high contiguity assembled genome efficiently [1]. At the time we performed the assembly, FlyE, WTDBG2, and MECAT2 were neither published nor available. MECAT is based on CANU and is also a correction-based assembler [2]. minimap2/miniasm is the predecessor of WTDBG2 and would collapse repeats that are longer than the input reads (https://github.com/lh3/miniasm). However, to compare assemblers that work on uncorrected reads, we assembled the 75x full dataset of NC358 using WTDBG2. The assembly statistics are summarized in Suppl. Table 2. This assembler generated a much more fragmented genome assembly (N50 of contigs at only 101 kb) compared to our Falcon-Canu hybrid approach and is also characterized by poor repeat assembly of the genome (LAI of 2.5). We had better success generating an assembly from the corrected reads using the FlyE assembler. Both contiguity (N50 = 2.3 Mb) and completeness (LAI = 21.0; Complete BUSCO = 96.4%) were very good using this approach but not as high as was possible with the Falcon-Canu pipeline (see summary statistics in Supp. Table 2). We report these data in our manuscript supplement but continue to base our summaries of assembly quality on results from the Falcon-Canu pipeline since this represents the best performing approach with our data and the complex structure of the maize genome.

=======

3. Recently the developer of Falcon released a new assembler called Peregrine based on minimizers of minimizers that works on high-quality PacBio reads (really meant for HiFi circular consensus reads but may work on the reads presented here). It would be extremely interesting to see how Peregrine performs on this Maize dataset; if not then a mention in the introduction or discussion concerning future developments. Since much has been done to compare many of these tools both in the literature and online across several species, the authors really need to place their findings squarely in the context of how this helps plant biologists beyond what is already known.

Response:

We ran Peregrine on the FALCON-corrected reads of the full 44x depth dataset. Peregrine was able to achieve an assembly contig N50 of 8.1 Mb and BUSCO completeness of 95.4% (C:95.4% [S:90.3%,D:5.1%], F:1.9%, M:2.7%, n:1440). By selecting the longest 23x reads from the 44x corrected reads, the assembly N50 was boosted to 11 MB (95.2% BUSCO completeness). In terms of the N50 metrics and contig number for maize, the FALCON-Peregrine hybrid assembly approach is not quite to the level of the FALCON-Canu hybrid assembly approach. The current version of the Peregrine assembler is mostly developed and tested on human datasets. Jason Chin (a co-author on this manuscript and developer of both Falcon and Peregrine) and colleagues will, in the future, investigate optimization of the SHIMMER indexing parameters for highly repetitive genomes like Maize. As the indexing schema and the initial design goals for Peregrine are quite different from Canu and FALCON, we think, and the reviewer also pointed out, it may distract the readers from the main goal of the paper, which is focusing on the impact of coverage and length on assembly metrics. We have added summary statistics for the FALCON-Peregrine hybrid assembly (based on the longest 23x corrected reads) in Suppl. Table 2 along with the other assembly methods that were initially tested and those that were added in response to the comment above.

==========

4. Two things in the text that were not clear and require some mention. First, the authors refer to the reads as "raw reads" in the text. Are these raw polymerase reads or subreads? In the methods it seems the authors are using "raw reads" in the body to mean subreads. This needs to be clear in the body. Second the authors mention the B73v4 and human PacBio datasets, and in there are some mention of them in the methods as well as supplement. The relevant data is in Table S2, and this is referenced once at line 72. Currently the relevance of adding this dataset is not clear in the body of the text; a sentence to highlight the comparison in Table S2 could make this clearer.

Response:

Thank you for pointing out these areas that need clarification. "Raw reads" in the text was referring to "subreads". We have changed this in the text to be subreads for consistency. We used the B73v4 dataset and the human PacBio dataset as two representations of data generated at different sequencing lengths (subread N50 of 16 kb and 11 kb, respectively), and also used the B73v4 dataset to test different assembly approaches for selection of the optimal approach for this study. These results are summarized in Suppl. Table 2 and described in the Methods section - Genome Assembly. We have also highlighted the relevance of the B73v4 assembly to our work in the main text and have rewritten the sentence describing how the

PacBio reads from the B73v4 and human datasets were used both to test assembly approaches and to guide our subsampling for mirroring empirical distributions.
==========

Minor points

5. Line 96: should it read dramatic read in contiguity since the reference is to contig N50?

Response:

Thanks for the suggestion. We have changed “dramatic drop in quality” to “dramatic drop in contiguity” in the text.
==========

6. Figure 1I: It is difficult to determine the number of SVs from the graph; would be nice to get a sense of number as well as size.

Response:

Thank you for this suggestion. We have added a new supplemental table (Suppl. Table 3) that contains this and additional metrics on the assembly variants detected by the optical map.
==========

7. Line133: Why only annotate two assemblies? Where the missing genes found in the gaps paralogs?

Response:

Based on BUSCO results (Figure 1D), RNA-seq mapping results (Figure 1G), and tandem gene array verification results (Figure 2G), we determined the 21k_20x and the 21k_75x assemblies were the two extremes of gene space assembly for NC358, and the other assemblies were similar to the 21k_75x assembly. However, further analyzing the *de novo* gene annotation results of these assemblies would be helpful to understand the gene space assembly. We mapped genes from the 21k_20x and 21k_75x assemblies to the other assemblies, and found these genes have similar mapping rate, suggesting the gene space assembly is similar except in the 21k_20x assembly. Furthermore, we found genes in the 21k_20x assembly contained more assembly gaps. Together with the large proportion (24.5%) of assembly gaps in 21k_20x assembly (Table 1), we show that the 21k_20x assembly has more fragmented gene space assembly and the large number of missing genes in this assembly were due to gaps. These new results were summarized in **Suppl. Table 7** and are described in the main text in lines 127-130.

==========

8. At what level are the heuristics of the assemblers impacting the results? For instance, is there a coverage threshold that makes the 20x coverage assembly break at some points? Since the authors of Canu are on the manuscript some mention of assembler strengths and limitations would be informative.

Response:

The recommended method to handle low coverage data in Canu is to modify parameters in the error correction step. However, since we use error-corrected reads from Falcon, the Canu

parameter for 20x coverage is identical to the rest of the experiments. The parameters for Falcon error correction on 20x coverage data can be found in our supplemental text. We have not observed a coverage threshold that specifically breaks down assembly at low coverage, but instead observed a general lack of sufficient reads to reconstruct the original genome as depth decreases. This was also observed in a study using both simulated and empirical long-read data to perform whole-genome assembly under various depths in Prokaryotes [3]. We have included the original figure from the paper below for ease of reference.

=======

9. If other assemblers were/are not tested, a justification of why only these long read assemblers were used would be good for the reader.

Response:
As the goal of this study is to explore the impact of different read lengths and coverage in the assembly of repetitive regions, we initially chose the most mature assemblers to perform the assembly instead of comparing all assemblers. There are numerous studies that have systematically compared assemblers (e.g., [4], [3], [5], [1], [6]). We have now cited these studies in our introduction to provide information for the reader. Additionally, as described above, we generated assemblies using WTDBG2, FlyE, and Peregrine with our 75x data set (both uncorrected and corrected) and found that our hybrid Falcon-Canu approach resulted in the most contiguous and complete assembly (results of these additional experiments have been summarized in Suppl. Table 2). Our manuscript summarizes performance at various sequence depths and lengths to guide future resource allocation questions and to inform the extent of biological questions that can be addressed from assemblies generated using variable input data. We agree it is important to use the optimal assembly approach to do this and have expanded the number of approaches that are tested in determining the optimal approach. However, we feel in the main body of the text it is best to focus on only the results of the best-performing method, in this case the Falcon-Canu hybrid approach, to not distract from the main purpose of this study.
=======

Reviewer #2 (Remarks to the Author):

This paper gives a detailed report of an in-depth evaluation on how to use long-read sequencing data to produce a high-quality assembly for a complex plant genome. Focusing on maize, the authors report assembly results using differently sized data sets and make a completeness assessment of genes, transposable elements, and highly repetitive features. Overall, the applied strategy and methods are sound but on some points, for example when discussing genic regions, the reported results are incomplete and as such do not offer detailed information on potential consequences for downstream biological data analysis (which is of major importance for most plant biologists). Whereas most of the results are in line with previous reports on long-read sequencing, this paper does describe a useful resource reference to the community.
Response:
Thank you for your insights. We provide below a point-by-point response.
=======

Major
1. The title and abstract are somewhat misleading, as all evaluated assemblies (Table 1) are not the result of solely assembling an increasing number of long reads sequences, they also contain information from optical maps and GoldenGate genetic markers to perform superscaffolding. As such, most of the reported results do not only deal with long read assembly, but include additional assembly strategies which also come at a cost. Related to terminology, if in the text the term superscaffolding is used, please also use this term in Table 1 (as from your text I conclude you report N50 stats on superscaffolds here).
Response:

We agree with the reviewer that the effects of our optical and genetic maps should be summarized as one of the main findings and have added this information to the abstract and strengthened this point in our conclusion. However, since the main goal of the paper is focused on the impact of read coverage and length and the optical map is held constant, we believe the current title effectively summarizes this focus. As for the terminology, we have changed "Scaffold N50" in Table 1 to "Superscaffold N50" as suggested by the reviewer.
=======

2. I am missing some key references which should be integrated to put the presented results in context. For example, the Belser et al paper (https://www.nature.com/articles/s41477-018-0289-4) generated and evaluated genome assembly using nanopore long reads for 3 different plant species. Likewise, Van Bel et al (https://www.ncbi.nlm.nih.gov/pubmed/30797187) gave a detailed overview of different data types and methods to assess completeness and actually performed a gene space comparison on different maize B73 assemblies. None of the presented results are discussed in light of these papers or other literature, which is extensive, on genome assembly strategies for complex plant/eukaryotic genomes.
Response:
Thank you for pointing out these two key references that we previously omitted. Belser et al. (2018) assembled three vascular plant genomes (two brassicas and one banana, 530 Mb - 630 Mb) that were sequenced using Nanopore. These authors observed a significant improvement of contiguity with these data compared to other plant genome assemblies (contig N50 > 5 Mb), and Bionano optical and genetic maps further assisted in constructing long scaffolds and chromosome-level assemblies. For assemblers, they tried Ra, SMARTdenovo, WTDBG, and Canu (not finished due to compute time) for all three genomes, and selected the best assembly. Ra generated the best assembly in general. They also found that the banana genome needed only the 30x longest reads to generate the most contiguous assembly, suggesting longer reads may be more valuable than higher depths.
Van Bel et al (2019) summarized changes in gene, isoform, and non-coding gene completeness scores (CEGMA, BUSCO) in nine model species and described the number of complete, fragmented, added, and removed genes in maize from v1 to v4 of the maize genome, providing suggestions for gene annotation practices. We have referenced this information in the introduction (lines 58-65) to provide readers with additional context.

=======

3. The evaluation of the genic regions (line 133-) reports some mapping statistics but lacks strong conclusions. Based on Figure 1G and Table S17, I learned that the RNA-Seq mapping rate for the NC358 tissue samples is higher for the B73 genome compared to the best NC358 assembly. This indicates some of the maize gene space present in B73 and NC358 is missing in the NC358 assemblies, though this is not discussed. Are these repeat-rich genes like disease resistant genes or are these genes located near centro/telomeres? It would be good to also differentiate between multimapped and non-mapping RNA-Seq reads on the NC358 assemblies. It would be informative of the authors can report how many and which type of genes these are missing in the NC358 assemblies, based on the B73 gene models recovered

by the NC358 RNA-Seq data, as this has major consequences for downstream gene-based anaslyses. Furthermore, taking the B73 gene models as a reference (though the Van Bel paper indicates >1000 of full-length genes present in V3 were removed in V4, which came as a surprise to me), how many of these genes are found back as full-length/partial, models in the NC358 assemblies? This would help to put the "obtained a total of 28,275 and 39,578 genes" (line 134) in perspective and could reveal if the best N50/contiguity assemblies also correspond with the assemblies best representing the maize gene space.

Response:
Thank you for pointing out the lack of clarity in the presentation of these two analyses. We mapped the NC358 RNA-seq libraries to the B73v4 genome solely for the purpose of checking the quality of the RNA-seq data. The data presented in Suppl. Table 19 contain both uniquely mapped and multi-mapped reads with an average mapping rate of 86.43%. In the main text, we only report the unique mapping rate of these RNA-seq libraries to NC358 assemblies, which is about 80%. If we also include the multimapped reads, it would be 86.97%. This is in concordance with the BUSCO score as reported in Suppl. Table 2, that the best NC358 assembly (full dataset Falcon+Canu) has slightly higher BUSCO scores (96.3%) compared to the best B73 assembly (94.9%), and is also consistent with these reads being mapped back to their genome of origin.

Please see response to Reviewer 1, point 7 for more details on this additional experiment. While we can provide functional annotation to the set of missing genes, we feel this would be misleading as these are random based on the subset of reads that were sampled and a different subset of genes will likely be missing in an assembly from a different subset of reads.

The annotation evidence and methods that were used for the V3 and V4 assemblies were different than those used in the two NC358 assemblies that we annotated. A comparison between B73 and NC358 is therefore not as appropriate of a comparison as is the comparison between the two NC358 annotations that use the same input evidence and same annotation methods. Furthermore, because the gene space in NC358 is distinct from B73, B73 annotations are not an appropriate point of reference for the completeness of genic regions in this tropical line. However, we have now taken our 75x NC358 annotations as a point of reference and have mapped these back to all of our lower depth/shorter read length NC358 assemblies, and have incorporated the excellent suggestion to characterize the extent to which these are found as full-length and partial sequences in these assemblies, to better characterize degradation of assemblies in genic regions.
=======

Minor
4. For the downsampling to lower coverages, every time only one sub-sample was selected, assembled and analyzed. Although this reviewer acknowledges that generating assemblies is resource intensive, it would be good to see, for one/few of the lower coverages, how big the

standard deviations are on the reported statistics in Table 1, when considering multiple sub-samples (e.g. n=5).

Response:
The downsampling was performed based on the random sampling of millions of reads. There is a chance that the sub-sampling process could pick a disproportionate number of poor reads and result in a poor assembly. There is also a chance that the sub-sampling process could pick, on average, better quality reads and result in a higher quality assembly. However, evidence suggests it is more likely that the small sample size (i.e., 20x) could not provide enough sequence information to reconstruct the original genome, as has been observed in a study based on simulated and empirical long-read data to test the effect of different depths of Prokaryote genome assemblies [3]. We have added text in our methods section (lines 253-259) discussing this point to the revised manuscript.

=======

5. If the aim of this paper is serving as a guide for future plant sequencing initiatives, adding a brief analysis of the costs associated with the different data types could be relevant.

Response: We appreciate this suggestion given the central question of this manuscript regarding resource allocation. However, sequencing technologies are under constant and rapid development to increase throughput and as such the cost to generate data is also rapidly changing. Furthermore, the "cost" of sequencing is highly variable between sequencing centers based on the degree of institutional subsidies. Thus, we feel adding in such information could be misleading.

=======

Reviewer #3 (Remarks to the Author):

This manuscript focuses on practical, technical concerns for the many biological researchers who are using next generation genomic sequencing technologies to advance their research interests. Getting accurate and comprehensive sequencing results from plant genomes is especially difficult. In this set of studies, the authors have compared different datasets for a strain of maize to help determine what characters are most important in providing good, useful assembled sequences according to different criteria. PacBio and Illumina sequencing platforms were used to provide hybrid assemblies of excellent quality. Further, they use BioNano chromosomal mapping to extend their results. By comparing the use of different PacBio read lengths, read-depths, and evaluating each for different applications, the authors provide very useful information for others seeking to make best use of their resources for accomplishing their individual goals. The presentation is well-written, clear, and effective in using graphs. The research is very detailed, even exhaustive and should be a great example for others wanting to take on the daunting task of sequencing a genome as difficult as many plant genomes are (including maize). The only question I have, and it is only an academic one is whether they had

thoughts or experience with respect to Oxford Nanopore technology, and whether their would be a value of including this platform for sequencing in cases where it seems (according to the current study) that read length seems most important to the quality of the results. The authors can address this if they wish. I think the manuscript should be accepted for publication. I came across no errors that needed to be corrected at this time.

Response:

Thank you for the nice summary regarding the methodologies as well as the research findings. As for combining the Oxford Nanopore technology with the Pacbio technology, our group tried this and produced exciting results, which are summarized in this preprint: Liu et al. (2020) Gapless assembly of maize chromosomes using long read technologies https://www.biorxiv.org/content/10.1101/2020.01.14.906230v2. In short, we found Pacbio data provides accurate assembly for the majority of the Ab10 maize genome, while the long-span Nanopore reads bridge the gaps at highly repetitive sequences, resulting in a much higher N50. These two studies complement each other, and support the reviewer's intuition that longer reads can further improve a maize genome assembly.

=======

References

1. Jayakumar, V. & Sakakibara, Y. Comprehensive evaluation of non-hybrid genome assembly tools for third-generation PacBio long-read sequence data. *Brief. Bioinform.* **20**, 866–876 (2019).
2. Xiao, C.-L. *et al.* MECAT: fast mapping, error correction, and de novo assembly for single-molecule sequencing reads. *Nat. Methods* **14**, 1072–1074 (2017).
3. Wick, R. R. & Holt, K. E. Benchmarking of long-read assemblers for prokaryote whole genome sequencing. *F1000Res.* **8**, 2138 (2019).
4. Kolmogorov, M., Yuan, J., Lin, Y. & Pevzner, P. A. Assembly of long, error-prone reads using repeat graphs. *Nat. Biotechnol.* **37**, 540–546 (2019).
5. Goldstein, S., Beka, L., Graf, J. & Klassen, J. L. Evaluation of strategies for the assembly of diverse bacterial genomes using MinION long-read sequencing. *BMC Genomics* **20**, 23 (2019).
6. Belser, C. *et al.* Chromosome-scale assemblies of plant genomes using nanopore long reads and optical maps. *Nat Plants* **4**, 879–887 (2018).

REVIEWERS' COMMENTS:

Reviewer #1 (Remarks to the Author):

The authors have addressed most of my concerns and comments.

Reviewer #2 (Remarks to the Author):

I thank the authors for the revised manuscript, which properly addresses my earlier comments. This manuscript will provide an important reference paper for the application of long-read sequencing and assembly of complex plant genomes.

Reviewer #3 (Remarks to the Author):

In my initial review of this manuscript, I thought that it was large, comprehensive, insightful, and very helpful addition to the craft of creating better assemblies of very complex plant genomes. I had no real concerns about the manuscript as it was. My fellow reviewers, having more current experience in this field, came up with some reasonable suggestions for improvement and clarification of the text. I think the authors have done a good job of addressing the reviewers' concerns, and in the process have improved the manuscript substantially. The authors have added some additional analyses, addressed specific concerns, clarified some of the language used, and provided a more comprehensive background in the introduction. I think the changes should make this manuscript very helpful to those deciding on what path to take in generating a genome assembly. Assuming that the other reviewers are largely satisfied with the responses, I feel there is no reason to hold back on publication at this time.

Roger Barthelson
